# Gut and oral microbiota associations with viral mitigation behaviors during the COVID-19 pandemic

**DOI:** 10.3389/fcimb.2022.966361

**Published:** 2022-09-09

**Authors:** Kelvin Li, Barbara A. Methé, Adam Fitch, Heather Gentry, Cathy Kessinger, Asha Patel, Vickie Petraglia, Pruthvi Swamy, Alison Morris

**Affiliations:** ^1^ Center for Medicine and the Microbiome, University of Pittsburgh School of Medicine, Pittsburgh, PA, United States; ^2^ Division of Pulmonary, Allergy and Critical Care Medicine, Department of Medicine, University of Pittsburgh School of Medicine and University of Pittsburgh Medical Center, Pittsburgh, PA, United States

**Keywords:** COVID-19, microbiome, ecological stability, saliva microbiota, gut microbiota, 16S rRNA gene amplicon sequencing

## Abstract

Imposition of social and health behavior mitigations are important control measures in response to the coronavirus disease 2019 (COVID-19) pandemic caused by the Severe Acute Respiratory Syndrome Coronavirus 2 (SARS-CoV-2). Although postulated that these measures may impact the human microbiota including losses in diversity from heightened hygiene and social distancing measures, this hypothesis remains to be tested. Other impacts on the microbiota and host mental and physical health status associations from these measures are also not well-studied. Here we examine changes in stool and oral microbiota by analyzing 16S rRNA gene sequence taxonomic profiles from the same individuals during pre-pandemic (before March 2020) and early pandemic (May-November 2020) phases. During the early pandemic phase, individuals were also surveyed using questionnaires to report health histories, anxiety, depression, sleep and other lifestyle behaviors in a cohort of predominantly Caucasian adults (mean age = 61.5 years) with the majority reporting at least one underlying co-morbidity. We identified changes in microbiota (stool n = 288; oral n = 89) between pre-pandemic and early pandemic time points from the same subject and associated these differences with questionnaire responses using linear statistical models and hierarchical clustering of microbiota composition coupled to logistic regression. While a trend in loss of diversity was identified between pre-pandemic and early pandemic time points it was not statistically significant. Paired difference analyses between individuals identified fewer significant changes between pre-pandemic and early pandemic microbiota in those who reported fewer comorbidities. Cluster transition analyses of stool and saliva microbiota determined most individuals remained in the same cluster assignments from the pre-pandemic to early pandemic period. Individuals with microbiota that shifted in composition, causing them to depart a pre-pandemic cluster, reported more health issues and pandemic-associated worries. Collectively, our study identified that stool and saliva microbiota from the pre-pandemic to early pandemic periods largely exhibited ecological stability (especially stool microbiota) with most associations in loss of diversity or changes in composition related to more reported health issues and pandemic-associated worries. Longitudinal observational cohorts are necessary to monitor the microbiome in response to pandemics and changes in public health measures.

## Introduction

The coronavirus disease 2019 (COVID-19) pandemic caused by the Severe Acute Respiratory Syndrome Coronavirus 2 (SARS-CoV-2) is a devastating worldwide event that has precipitated dramatic changes in social and health behaviors in human populations (Atzrodt et al., 2020). Especially in the early pandemic phase in 2020 prior to vaccine and other pharmaceutical prophylaxis interventions, a variety of strategies were implemented to minimize the spread of the virus including social distancing, self-isolation, working from home and increased hygiene measures (Bavel et al., 2020). Substantial efforts have been underway to understand the impacts of these disruptions and COVID-19 related worries on human psychology including stress, and anxiety (Blix et al., 2021), as well as health consequences such as changes in diet, sleep, and exercise (Arora and Grey, 2020). Several, longitudinal studies assessed mental health outcomes within the same individuals before and during the pandemic and determined that general mental distress increased during the pandemic (Daly et al., 2020; Pierce et al., 2020) and effects of COVID-19 on daily life were significant predictors of higher levels of depression, anxiety, and stress during the pandemic (Haliwa et al., 2021). However, the impact of these population-wide viral transmission minimization strategies and other behavioral changes on the human microbiota of individuals non-symptomatic for COVID-19 have not been well-studied despite the substantial and complex interplay between diet, environment factors and the microbiome in human health and disease.

The human microbiota and its collection of genomes (the microbiome) is composed of trillions of cells that interact as microbial communities in multiple ecological niches in and on the human body through mutualistic or symbiotic relationships with the host (Gevers et al., 2012; Sender et al., 2016). More than a decade of research has underscored the multiple, critical roles the microbiome plays in normal development and maintenance of the immune, endocrine, and nervous systems, and healthy metabolism.

As the COVID-19 pandemic has progressed, several groups have speculated on the potential impact of these changes in behavior and lifestyle on the microbiome (Domingues et al., 2020; Burchill et al., 2021; Finlay et al., 2021). In particular, it has been hypothesized that these changes may include the loss of microbial diversity due to increased microbial depletion and reduced transmission resulting from heightened hygiene and social distancing measures, respectively (Domingues et al., 2020; Burchill et al., 2021; Finlay et al., 2021). However, this hypothesis has not been well-studied longitudinally in individuals not infected with COVID-19. Here we used an ongoing large observational cohort (MedBio Cohort) with pre- and early pandemic data and stool and oral specimens from the same individuals to examine associations between human microbiota and changes in social behaviors precipitated during an ongoing pandemic and concomitant changes in public health measures.

## Materials and methods

### Cohort

Participants were selected without bias from a large ongoing observational cohort (MedBio) consisting of a collection of UPMC patient registries and clinical investigations which facilitates standardized approaches to subject enrollment and specimen processing across multiple studies. These studies span a range of chronic illnesses and disease status. The University of Pittsburgh IRB approved the study, and all participants signed informed consent.

### Sample collection

Early pandemic samples were collected from May 2020 through November 2020. Stool specimens were self-collected using the DNA/RNA Shield Fecal Collection tubes (Zymo) for nucleic acid preservation and short-term (two to four weeks) storage at ambient temperature. Oral specimens were self-collected using the OMNIgene·ORAL OM-505 devices. 2 mL of saliva were collected for nucleic acid preservation and short-term storage at ambient temperature. Specimens were mailed to the University of Pittsburgh. Upon receipt, specimens were sub-aliquoted prior to long-term storage at -80°C.

### Early pandemic data collection

During the early pandemic period, subjects participated in an on-phone interview assessing demographics, medical history, smoking history, health status, and COVID-19-related behavior with specimen collection occurring within approximately 10-14 days of the interview. We also administered the General Anxiety Disorder 7 (GAD7) questionnaire (Johnson et al., 2019), Patient Health Questionnaire 9 (PHQ-9) questionnaire (Kroenke et al., 2001), and the Insomnia Severity Index (ISI) (Morin et al., 2011).

The questionnaire consisted of 49 grouped questions: Demographics (Q1-Q6), Past Health History (Q7-Q12, Q12 was an inventory Q12a-Q12q of comorbidities), Smoking History (Q13-Q15), Recent Heath History (Q16-Q19), GAD7 Anxiety (Q20), PHQ-9 Depression (Q21), ISI Sleep Survey (Q22), and Recent Behavior (Q23-Q49). Some questions were excluded from the statistical analyses as they were either regarding the accessibility of UPMC medical resources, or the potential relevance of the question was more directly represented by an alternative question.

Categorical responses were recoded into ordinal responses when necessary, so that 0 (reference) was associated with healthy or no difference. Responses to inventory-style questions were summed up (Q17 General Ailments). (See Supplemental material for a PDF version of the questionnaire.) See **Table 1**. “Questionnaire Summary Table” for a summary of descriptive statistics for the subset of responses included in the models.

**Table 1:** Questionnaire Descriptive Statistics Summary.

	Variable	Categories	Mean	(95% CI: LB, UB)	[N]	Question ID
1.)	Age		61.505	(60.285, 62.726)	[582]	Q1
2.)	Sex	*Male*	0.455	(0.415, 0.497)	[266]	Q2
		*Female*	0.545	(0.503, 0.585)	[318]	
3.)	Ethnicity	*Black*	0.068	(0.049, 0.091)	[40]	Q3
		*Other*	0.087	(0.065, 0.112)	[51]	
4.)	Education Level ^1^	*range [0, 5]*	2.642	(2.538, 2.746)	[586]	Q5
5.)	Health ^2^	*range [0, 4]*	2.342	(2.265, 2.42)	[587]	Q7
6.)	Fever (past year)	*Yes*	0.128	(0.101, 0.158)	[72]	Q10
7.)	Exercise Pre-Pandemic (>1x/week)	*Yes*	0.642	(0.602, 0.681)	[377]	Q11
8.)	High Blood Pressure	*Yes*	0.453	(0.412, 0.494)	[265]	Q12a
9.)	Diabetes	*Yes*	0.116	(0.091, 0.145)	[68]	Q12b
10.)	Sleep Apnea	*Yes*	0.193	(0.162, 0.228)	[113]	Q12h
11.)	Asthma	*Yes*	0.156	(0.127, 0.188)	[91]	Q12j
12.)	Cancer (active treatment)	*Yes*	0.070	(0.051, 0.094)	[41]	Q12l
13.)	Immune System Disease (excluding HIV)	*Yes*	0.281	(0.245, 0.319)	[164]	Q12n
14.)	Smoking History	*Yes*	0.502	(0.46, 0.543)	[293]	Q13
15.)	Sum of Ailments	*range [0, 10]*	1.053	(0.914, 1.191)	[588]	Q17
16.)	GAD7 Anxiety Score	*range [0, 21]*	2.827	(2.512, 3.141)	[588]	Q20
17.)	PHQ9 Depression Score ^3^	*range [0, 24]*	2.621	(2.33, 2.912)	[588]	Q21
18.)	ISI Sleep Survey	*range [0, 28]*	11.316	(10.928, 11.705)	[588]	Q22
19.)	Exercise Early Pandemic (>1x/week)	*Yes*	0.708	(0.668, 0.745)	[402]	Q26
20.)	Sum of COVID-19 Worries	*range [0, 15]*	5.184	(4.958, 5.409)	[588]	Q38, Q39, Q41
21.)	Social Distancing ^4^	*Yes*	0.930	(0.907, 0.95)	[547]	Q42
22.)	Change in Diet ^5^	*range [0, 4]*	0.683	(0.605, 0.762)	[584]	Q46
23.)	Number Cohabitants ^6^	*range [0, 3]*	1.434	(1.338, 1.531)	[587]	Q47
24.)	Number of Pets ^6^	*range [0, 3]*	0.920	(0.834, 1.005)	[587]	Q48

^1^Education Level coding: 0 = “did not graduate from high school” to 5 = “doctorate”.

^2^Health coding: 0 = “poor” to 4 = “excellent”.

^3^Last PHQ9 item (suicide) was omitted from questionnaire.

^4^Either Social Distance or Working From Home.

^5^Coding: 0 = “not at all” to 4 = “ a lot”.

^6^Coding: 0 = “none” to 3 = “3 or more”.

This table contains a summary of the variables included in the analyses that were based on the questionnaire responses. The category or range of values, mean, 95% CI, N, and question identifier have been reported for each variable. Note that the subset of subjects that were used in the stool or saliva analyses depended on matching samples. This table represents the descriptive statistics from all the questionnaire responders, regardless of whether they could be included in the stool or saliva samples analyses.

### Other subject information

BMI was estimated by linear interpolation using the closest bracketing BMI measurements taken before and after the collection date of the sample. Samples taken before March 15, 2020, were considered pre-pandemic samples, and matching samples from the same subject collected after March 15, 2020, were considered early pandemic samples (when “lockdown” and more intense viral transmission mitigation strategies were in place). Early pandemic samples were collected from May 2020 through November 2020.

### DNA extraction

DNA extraction was performed using the Qiagen Powersoil Microbiome Kit EP for automated DNA extraction using an Eppendorf, 5075VTC liquid handling workstation. HEPA filtration was used during sample processing and the workstation was UV sanitized between batches. Specimens were processed per manufacturer’s protocol with the following modifications: An approximate aliquot of 300μl of specimen was added to individual bead beating tubes to ensure no carryover between samples during the bead beating process. Aliquots from the individual tubes were then transferred to 96-well blocks for completion of the automated genomic DNA extraction process. Reagent blanks were included as negative controls. Cells and genomic DNA from a microbial community of known composition (ZymoBiomics Microbial Community Standards; Zymo Research, Irvine, CA) served as positives controls. As a component of the QC process, positive controls were evaluated across sample batches to evaluate laboratory and sequencing performance and compared to historical performance of 16S rRNA gene sequencing at the Center for Medicine and the Microbiome (CMM). No significant batch deviation was identified in this project.

### Bacterial community sequencing

Extracted genomic DNA (gDNA) was amplified for the V4 region using Q5 HS High‐Fidelity polymerase (New England BioLabs, Ipswich, MA) with inline barcode primers design based on the method of Caporaso (2012) (Caporaso et al., 2012). V4 primer sequences were: 515f 5’-GTGCCAGCMGCCGCGGTAA-3’ and 806r 5’-GGACTACHVGGGTWTCTAAT-3’. Approximately 5-10 ng of each sample were amplified in 25 µL reactions. Cycle conditions were 98°C for 30 seconds, then 30 cycles of 98°C for 10 seconds, 57°C for 30 seconds, and 72°C for 30 seconds, with a final extension step of 72°C for 2 minutes. Amplicons were purified with AMPure XP beads (Beckman Coulter, Indianapolis, IN) at a 0.8:1 ratio (beads:DNA) to remove primer dimers. Eluted DNA was quantitated on a Qubit fluorimeter (Life Technologies, Grand Island, NY). Sample pooling was performed on ice by combining 40 ng of each purified band. For negative controls and poorly performing samples, 20 µL of each sample was used. The sample pool was purified with the MinElute PCR purification kit (Qiagen, Germantown, MD). The final sample pool underwent 2 more purifications: AMPure XP beads to 0.8:1 to remove primer dimers, and a final cleanup in Purelink PCR Purification Kit (Life Technologies). The purified pool was quantitated in triplicate on the Qubit fluorimeter prior to sequencing.

The sequencing pool was prepared as per Illumina’s recommendations (Illumina, Inc., San Diego, CA), with an added incubation at 95°C for 2 minutes immediately following the initial dilution to 20pM. The pool was then diluted to a final concentration of 7pM + 20% PhiX control (Illumina). Sequencing was done on an Illumina MiSeq 500‐cycle V2 kit (Illumina).

### Bioinformatics

Sequences from the Illumina MiSeq were deconvolved and then processed through the CMM in‐house sequence quality control pipeline, which includes dust low complexity filtering, quality value (QV<30) trimming, and trimming of primers used for 16S rRNA gene amplification, and minimum read length filtering. Using the scripts fastq_quality_trimmer and fastq_quality_filter from Hannon’s Cold Spring Harbor Laboratory’s FASTAX-Toolkit (http://hannonlab.cshl.edu/fastx_toolkit/). Reads were trimmed until the QV was 30 or higher. Trimmed reads shorter than 75bp or those with less than 95% of the bases above a QV of 30 were discarded. Forward and reversed paired reads were merged with a minimum required overlap of 25 bp, proportion overlap mismatch > 0.2, maximum N’s allowed = 4, and a read length minimum of 125 bp. Forward and reverse reads were merged into contigs then processed through the CMM’s Mothur‐based (v1.44.1) (Schloss et al., 2009) 16S rRNA gene sequence clustering and annotation pipeline. Sequence taxonomic classifications were performed with the Ribosomal Database Project’s (RDP) naïve Bayesian classifier (Wang et al., 2007) (Quast et al., 2013) with the SILVA 16S rRNA database (v138) (Quast et al., 2013).

### Data analysis

*Questionnaire Analyses.* Selected recoded questionnaire responses (variables), p = 25, were tested with the Shapiro-Wilk test for normality (H_0_: values are normally distributed). If the p-value was >0.2, then the original values were utilized. If the p-value <0.2, the values were then logarithm transformed and retested. If the transformation increased the p-value, then the transformation was accepted. Pearson correlations were calculated between responses and a Principal Component Analysis (PCA) was performed. Principal Components (PCs) which represented at least 5% of the total variance were then annotated by identifying the variable with the greatest correlation with the PC. Correlations were reported for those with p-values < 0.001 after a Bonferroni adjustment, assuming the number of tests were m = p(p-1)/2 = 300.

Due to the compositional nature of the taxonomic profiles from 16S rRNA gene sequencing (Gloor et al., 2224), taxonomic abundances were first transformed using the additive log ratio (ALR) transformation (Tarabichi et al., 2015). The top 15 taxa, by average abundance across the experimental samples, were selected to represent the taxa of interest, and the remaining taxa were accumulated into the denominator of the ratio, prior to natural log transformation. Log ratio transformations are crucial when including multiple taxa into linear models to ensure the abundances are normally distributed and independent from each other (Aitchison, 1982).

Analyses involving the calculation of a diversity index utilized the Shannon diversity index and the Tail statistic (Li et al., 2012). The Tail statistic is more sensitive towards the lower abundance taxa than the Shannon diversity index. Analyses requiring the calculation of pair-wise compositional distances between samples used the Manhattan distance, which is also more sensitive towards differences in the lower abundance taxa than the Euclidean distance.

### Adjusting for differences in sample collection times

There was a wide range of timespans between the pre- and early pandemic samples. The median and 95% Prediction Intervals (PI) time spans for stool were 526 (164, 1094) days and for saliva, 725 (251, 1046) days. Preliminary analyses to identify a corrective adjustment suggested that over time, paired sample distances approached a limit asymptotically. A non-linear adjustment for each timespan *t* based on fitting 3 parameters (maximum distance *m*, rate *r*, slope *s*) with the function: adjustment(t) = m*(1-exp(-t*r))+s*t) was calculated, but model comparisons revealed the adjustment was not significantly better than the simpler linear model with an intercept. This is likely due to the lower bound of the timespans being restricted between 5-6 months, so any acute changes to the microbiota composition might have already reached their limits. The correlation between changes in the stool and saliva from the same subject were also examined, but the near 100% correlation of the pre-pandemic to early pandemic timespans between the two sample types confounded the analysis.

### Models

Three statistical models were used to associate the microbiota sampling with the questionnaire responses.

The “pre/early pandemic paired” (PEPP) model first identified subjects with both pre-pandemic and early pandemic samples, then used the variables of days pre-pandemic, days early pandemic, pre-BMI, and dBMI (change in BMI) and questionnaire responses to predict the difference between the diversity, abundance or the distance (Stapleton et al., 2021) between pre-pandemic and early pandemic samples with a linear model.

The “pre/early pandemic cluster transition” (PEPCT) model, another type of paired analysis, used a combination of clustering and then logistic regression to associate questionnaire responses to changes in “microbiome type”. Pre- and early pandemic samples were first hierarchically clustered together, then clusters were iteratively identified by increasing cuts (*k*). At each cut *k*, for each of the resultant clusters identified from 1:*k*, the following variables were used to predict the sample’s early pandemic cluster: questionnaire responses, pre- and early pandemic days, pre-pandemic BMI and dBMI, and pre-pandemic cluster identifiers. This analysis identified factors that could predispose a subject’s sample to change to a specific early pandemic cluster (“arrive”). Similarly, a “departure” analysis was performed for each of the pre-pandemic clusters at each cut *k*. For each pre-pandemic cluster, member subjects were divided into those that remained in the same early pandemic cluster and those that departed. In the departure analysis, dBMI, pre- and early pandemic days, and the questionnaire responses were included in the logistic regression to predict whether a sample stayed in or left the pre-pandemic cluster. To identify which cluster cut *k* to report arrival or departure associations with, the cut *k* with the most significant p-value for each factor was selected. Taxonomic members (cluster unifiers) that differentiate a cluster from other clusters generated at the same cut, were identified with an R^2^ ratio analyses (See Supplemental methods). The R^2^ ratio analyses estimate the R^2^ between two clusters with (full model) and without (reduced model) a taxon of interest, to identify whether the taxon of interest contributed to cluster separation. If the reduced model had a smaller R^2^ than the full model, then the taxon left out of the reduced model contributed to cluster separation. Taxa that consistently contributed to a cluster’s separation from the other clusters were considered taxa that defined a cluster’s microbiota “type”.

The “early pandemic cross-sectional” (EPCS) model focused on the early pandemic samples. The questionnaire responses, EP-BMI, and days early pandemic were used to predict the microbiota diversity, inter-sample distancing, or abundance with a linear model.

**Figure 1:**
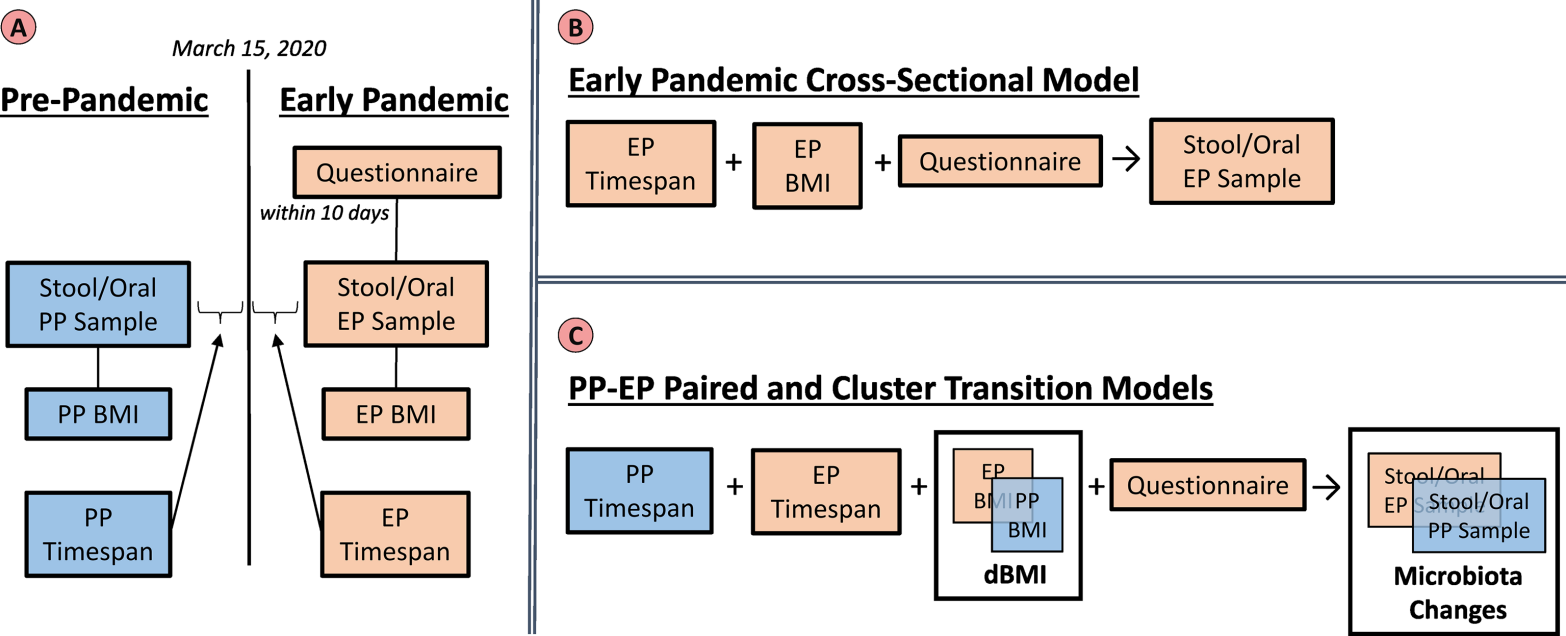
Variables and Models. The left panel **(A)** summarizes the groups of variables that were utilized in the analyses. Pre-pandemic (PP) (blue) and Early Pandemic (EP) **(beige)** variables include the taxonomic profiles from sequencing microbiota samples, BMIs interpolated based on the sample collection dates, and timespans relative to March 15, 2020. The questionnaire responses were only collected during the early pandemic. The top right panel **(B)** illustrates the early pandemic cross-sectional model. Here, only the EP variables: timespan, BMI, and questionnaire were utilized to build a model to predict the EP stool or saliva microbiota. The lower right panel **(C)** represents the variables included in the paired and cluster transition models. Both the PP and EP timespans, as well as questionnaire responses were included in the models. The BMI and microbiota profiles were included in the model as their relative changes which could be calculated per subject.

See **Figure 1**, “Variables and Models” for a diagram illustrating the relationship between variables and the models that were fit.

## Results

### Questionnaire

From the 588 questionnaire responders, the mean respondent’s age was 61.5 years old, although 95% of the subjects were between 25 and 82 years. 54.5% of respondents were female and 84.5% were Caucasian. The percent of respondents with a smoking history (>100 cigarettes in their lifetime) was 50.2%. The mean and 95% CI of the questionnaire responses can be found in **Table 1**, “Questionnaire Descriptive Statistics Summary”. The breakdown of respondents included in the pre-pandemic to early pandemic time points (PEPP), pre-pandemic to early pandemic cluster transition (PEPCT), and early pandemic cross-sectional (EPCS) models for stool and saliva analyses can be found in **Supplemental Table 1**, “Sample Exclusions”.

Examination of the correlation matrix with Bonferroni adjusted p-values < 0.001 identified several noteworthy correlations. See **Supplemental Figure 2**, “Questionnaire Response Correlations, Bonferroni Adjusted Significant (p-value < 0.05)” for the heat map and all pairwise correlations. Education level was correlated with health (ρ = 0.23) and exercise (>1x/week, pre-pandemic ρ = 0.29, early pandemic ρ = 0.25). Exercise pre-pandemic was correlated with early pandemic exercise with a coefficient of ρ = 0.6. A clustering of positive correlations was also identified among immune system disease, asthma, (sum of) general ailments, GAD7 anxiety, PHQ9 depression, and ISI sleep. (Sum of) COVID-19 worries was correlated with diet change (ρ = 0.24). The number of cohabitants was correlated with the number of pets (ρ = 0.24). Principal Components Analysis (PCA) revealed that the top 5 PCs each captured greater than 5% of the total variance, but the first 21 (out of 25) PCs would be required to capture 95% of the variance. The top 5 PCs were most closely correlated with: PHQ9 depression (ρ = 0.79), exercise (pre-pandemic) (ρ = 0.58), ethnicity (ρ = 0.67), exercise (early pandemic) (ρ = 0.45), and high blood pressure (ρ = 0.48).

### Statistical analyses of microbiota from stool and oral samples

Here results are reported first for the analyses of microbiota from stool then for saliva samples. For each sample type, the paired analysis results for the pre- and early pandemic samples are reported first followed by the cross-sectional results for the early pandemic sample analysis. Both paired and cross-sectional analyses are comprised of a collection of independent sub-analyses that fit statistical models using calculated sample diversity, inter-sample distances, and taxonomic abundances.

### Stool specimens for 16S rRNA gene sequencing

The sample size available for stool was 311 samples available for the early pandemic cross-sectional analysis and 288 subjects had both pre- and post-pandemic samples for the paired analyses.

### The pre/early pandemic paired analysis of stool sample microbiota

The pre/early pandemic paired (PEPP) analysis of stool sample microbiota was performed on the n = 288 paired pre- pandemic and early pandemic stool samples taken from the same subject. The median and 95% Prediction Interval (PI) of the pre- and early pandemic days were 342 (25, 912) and 162 (118, 223), respectively. These differences were then associated with the questionnaire responses from these subjects. The number of subjects with both samples available were fewer, so there was less statistical power than the EPCS stool analysis. Overall composition for the pre-pandemic and early pandemic stool samples can be found in **Supplemental Figure 1**, “Paired Compositional Stacked Bar Plots”.

*Diversity was stable between time points.* When examining stool microbiota, the Shannon diversity index did not identify any significant (p<0.05) associations, but the Tail statistic identified a negative association with change in BMI (p−val = 0.0232) and smoking history (p−val = 0.0317). The number of pets was positively associated with an increase in diversity (p−val = 0.0406). The intercept, representing the difference between pre- and early pandemic differences in diversity when controlling for other factors included in the model, was not significantly non-zero for neither the Tail statistic (β_0_ = -0.7441, p-val = 0.7341) nor the Shannon diversity index (β_0_ = −0.4881, p-val = 0.2297). The increase of BMI of subjects with paired stool samples was not statistically significant in the subjects: median dBMI = 0.0, 95% PI = (-4.326, 3.761).

**Figure 2:**
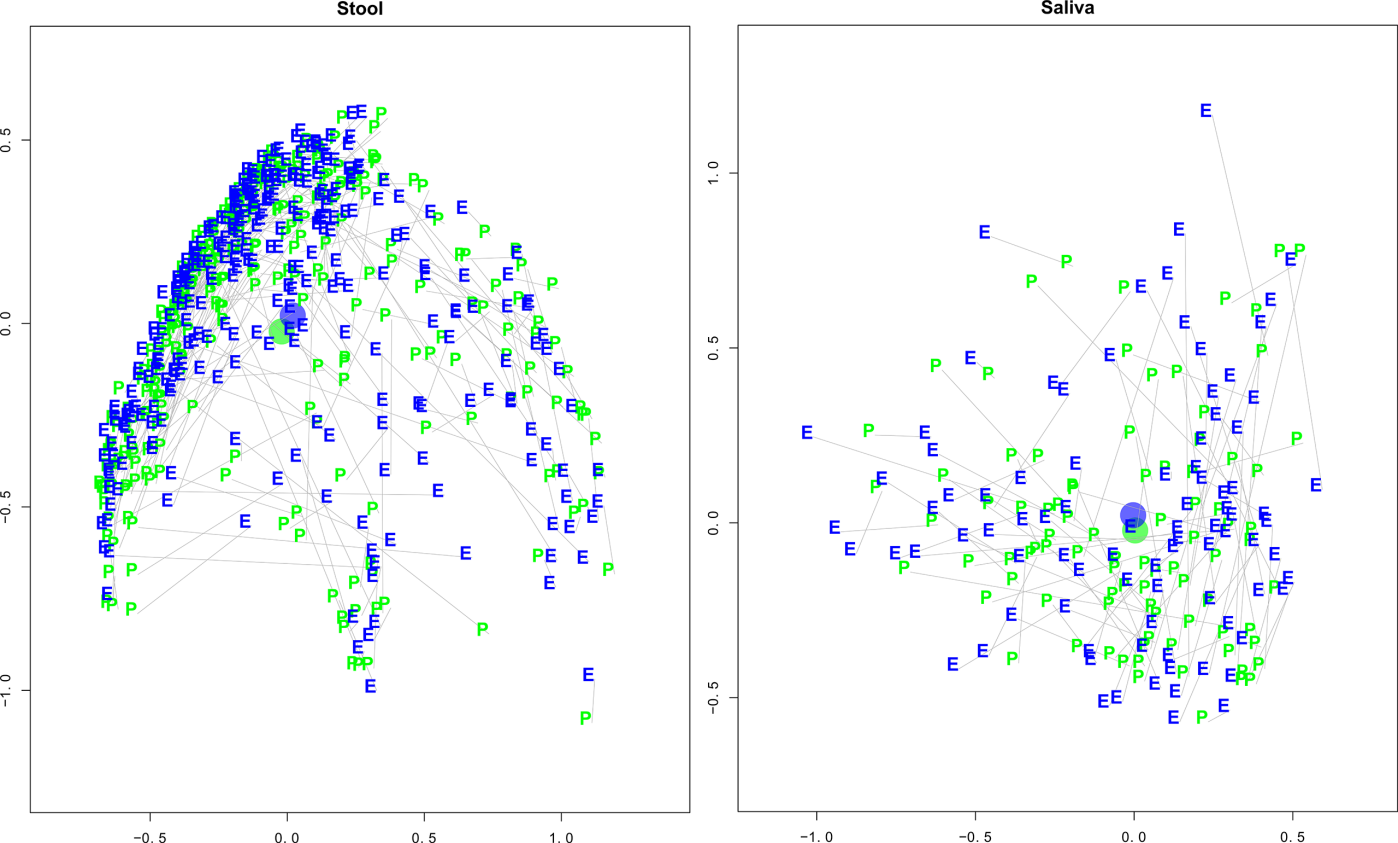
Stool and Saliva Pre- and Early Pandemic Paired MDS plots. These multi-dimensional scaling (MDS) plots illustrate each subject’s taxonomic compositional similarity between pre- and early pandemic samples in context with the samples of the cohort. The left and right panels represent the intra-cohort separation of stool and saliva samples, respectively. The green “pre” and blue “early” labels indicate the MDS estimated locations of pre- and early pandemic samples, respectively. A grey line connects pre- and early pandemic samples from the same subject. The blue and green circles represent the centroids of the pre- and early pandemic samples. After controlling for questionnaire responses, the bootstrapped regression identified that pre- vs early pandemic samples had a statistically significant separation (coef = 1.1404, p-val < 0.0001), but saliva did not (coef = 0.1421, p-val = 0.8461).

*BMI and health were associated with reduced pre-to-early pandemic inter-sample distances.* An analysis of the distance (compositional change) between stool sample pairs found a significant effect of lengthening the distance between pairs (greater change in composition) for pre-pandemic days (p−val = 0.0023) and days into the early pandemic (p−val = 0.0265). See **Figure 2**, Stool and Saliva Pre- and early pandemic MDS Plots. Pre-pandemic BMI (p−val = 0.0328) and health (p−val = 0.0178) had an effect of shortening the distance between pairs (composition becomes more alike). There was a less significant, but potentially noteworthy, effect of diabetes (p−val = 0.0708) and number of cohabitants (p−val = 0.0907).

*Changes in multiple taxonomic abundances were associated with immune system disorders and changes in BMI.* The number of days from stool sample collection to the early pandemic period were positively associated with two taxa. The number of pre-pandemic days before sample collection was associated with *Fusicatenibacter* (p−val = 3.33×10^-4^), and the number of early pandemic days before sample collection was associated with *Lachnoclostridium* (p−val = 5.13×10^-4^). Immune system disease was associated with the increase of *Alistipes* (p−val = 3.8×10^−5^), *Lachnospiraceae_uncl* (p−val = 1.02×10^-3^), *Bacteroides* (p−val = 1.08×10^-3^), and *Faecalibacterium* (p−val = 5.72×10^-3^). Asthma was associated with an increase of *Ruminococcus* (p−val = 8.12×10^-4^). Pre-pandemic BMI was associated with an increase in *Prevotella* (p−val = 3.64×10^-4^), and changes in BMI between sample collection dates were associated with less *Oscillospiraceae UCG_002* (p−val = 3.84×10^-4^) and *Subdoligranulum* (p−val = 4.58×10^-3^), and more *Escherichia_Shigella* (p−val = 4.55×10^-3^). Diabetes was associated with more *Agathobacter* (p−val = 9.89×10^-3^) in the early pandemic. In addition, there were associations with depression including a decrease in *Lachnospiraceae_uncl* (p−val = 2.46×10^-3^) and an increase of *Prevotella* (p−val = 8.78×10^-3^) with education level in the early pandemic period.

### Pre/early pandemic cluster transition stool analysis identified changes of cluster membership associated with sex, COVID-19 worries, asthma, cancer, and social distancing

A Pre/Early Pandemic Cluster Transition (PEPCT) stool analysis was performed to identify factors that are associated with samples changing their cluster membership between pre- and early pandemic time points. “Departer” samples were defined as those samples that left their starting pre-pandemic cluster for another cluster by their early pandemic time point. “Arriver” samples were defined as those samples that were new additions to a cluster in the early pandemic time point. When the hierarchical clustering was cut to *k* = 3 clusters the departers from the second cluster (*cl* = 2 of *k* = 3) consisted of fewer females (p-val < 0.001). Based on cluster influencer analysis, the distinguishing taxonomic member of this cluster was *Bacteroides*. When the hierarchical clusters were cut to *k* = 6, the departers from the second cluster (*cl* = 2 of *k* = 6) were associated with more COVID-19 worries (p-val = 0.006). The distinguishing members of this cluster were *Akkermansia Oscillospiraceae UCG_002, Bacteroides, Alistipes, Prevotella*, and others. Clusters with arriving samples with significant associations included (*cl* = 3 of *k* = 6) that were associated with fewer COVID-19 worries (p-val < 0.001). This cluster was distinguished by *Bacteroides*, *Faecalibacterium*, and *Agathobacter*. Samples from asthma subjects were associated with arrival in (*cl* = 1 of *k* = 2) (p-val = 0.009). The distinguishing taxa of this cluster were *Prevotella* and *Prevotellaceae*_uncl. Samples from subjects with cancer, arrived at (*cl* = 2 of *k* = 5) (p-val = 0.005) which was distinguishable by *Akkermansia*, *Oscillospiraceae UCG_002*, *Prevotella*, *Bacteroides*, *Escherichia_Shigella*, and others. Arrivers in cluster (*cl* = 1 of *k* = 2) were positively associated with social distancing (p-val = 0.009). See Supplemental Materials for additional descriptions and figures supporting this analysis. Clusters were considered from *k* = 2 to *k* = 6, after which individual clusters sizes became too small to associate factors with.

### Early pandemic cross-sectional analysis for stool

The early pandemic cross-sectional (EPCS) for stool focused on identifying associations between questionnaire responses and changes in the microbiota, while controlling for age, sex, and days into the early pandemic.

*Differences in diversity were associated with immune system disease and age.* A decrease in diversity was found in association with immune system disease (Tail: p−val = 3.69 ×10^−8^; Shannon: p−val = 1.83×10^−6^). Age was associated with an increase of diversity (Tail: p−val = 0.000263; Shannon: p−val = 0.000301). At less significance, health was associated with increased diversity (Tail: p−val = 0.0246; Shannon: p−val = 0.0282). GAD7 Anxiety was associated with increased Shannon diversity (p−val = 0.0676). Pre-pandemic exercise was associated with increased diversity (Tail: p−val = 0.0886; Shannon: p−val = 0.0689).

*Effects on Inter-sample distance were small, but associated with BMI, age, health, sex, and immune system disease.* EPCS analysis of stool microbiota using PERMANOVA revealed a number of significant associations, although all the effect sizes were relatively small with the greatest R^2^ at 0.0148 for days into the early pandemic (p-val = 3.704×10^−5^), followed by BMI (p−val = 1.5925×10^-3^), age (p−val = 3.704×10^-4^), health (p−val = 5.185×10^-04^), female (p−val = 3.704×10^−5^), immune system disease (p−val = 3.704×10^−5^), social distancing and working from home (p−val = 6.2738×10^-2^).

*Differences in taxonomic abundances were associated with sex, age, immune system disease, and BMI.* The EPCS stool microbiota analysis using taxonomic abundance as a response identified many significant associations (p-values < 0.001). These included associations with sex (female): *Prevotellaceae uncl* (negative, p−val = 2.84×10^−10^), *Prevotella* (negative, p−val = 2.31×10^-4^), and *Bacteroides* (p−val = 7.91×10^-4^); an association with Age: *Alistipes* (p−val = 8.09×10^−6^); immune system disease: *Oscillospiraceae UCG_002* (negative, p−val = 9.17×10^−6^), *Subdoligranulum* (negative, p−val = 1.57×10^−5^), *Ruminococcus* (negative, p−val = 1.62×10^-4^), *Lachnospiraceae_NK4A136_grp* (negative, p−val = 6.28×10^-4^), and *Fusicatenibacter* (negative, p−val = 8.06×10^-4^); days into the early pandemic: *Prevotella* (negative, p−val = 1.58×10^-4^) and *Prevotellaceae_uncl* (negative, p−val = 4.88×10^-4^) and with BMI: *Bacteroides* (p−val = 6.24×10^-4^) and *Lachnoclostridium* (p−val = 6.46×10^-4^).

### Saliva specimens for 16S rRNA gene sequencing

The sample size available for saliva was 218 early pandemic cross-sectional subjects and 89 subjects for the pre/early pandemic paired analyses. The decrease of BMI of subjects with paired saliva samples was not statistically significant: median dBMI = 0, 95% PI = (-2.434, 3.002). The median and 95% Prediction Interval (PI) of the pre- and early pandemic days were 520 (55, 907) and 172.5 (117,220), respectively.

### The pre/early pandemic paired analysis for saliva

The Pre/Early Pandemic Paired (PEPP) analysis for saliva samples identified fewer significant associations (p-value < 0.1) than the stool samples. Overall composition for the pre-pandemic and early pandemic saliva samples can be found in **Supplemental Figure 1**, “Paired Compositional Stacked Bar Plots”.

*Diversity was stable between time points.* While multiple associations were identified between saliva microbiota and diversity, only the positive association with immune system disease when measured by the Tail statistic (p−val = 0.0116) was strong. Marginally significant associations (p-value < 0.10) were also found with the Tail statistic that could corroborate significant associations found in other analyses: education level (negative, p−val = 0.06866), depression (p−val = 0.071907), number of cohabitants (negative, p−val = 0.097342), and anxiety (negative, p−val = 0.098607). The intercept was not significantly non-zero for Tail (β_0_ = −3.875, p-val = 0.1759) nor the Shannon diversity index (β_0_ = −0.9160, p-val = 0.147).

*Pre-to-early pandemic inter-sample distances were marginally associated with social distancing and COVID-19 worries.* The paired distance analysis using saliva samples identified a positive association with social distancing (p-val = 0.0262) and to a lesser extent, a negative association with COVID-19 worries (p-val = 0.0754).

*COVID-19 worries associated with Oribacterum and Campylobacter abundances.* Increased taxonomic abundances of saliva microbiota were associated between COVID-19 worries and *Oribacterium* (p−val = 0.00299) and *Campylobacter* (p−val = 0.00853).

The cluster transition analysis did not yield any significant associations with p-val < 0.01.

### Saliva cross-sectional analyses

*Associations with diversity were marginal except for BMI.* The EPCS saliva microbiota had marginal associations (p-values < 0.1) with diversity. Early pandemic BMI was associated with increased diversity (Tail: p−val = 0.015042; Shannon: p−val = 0.01897). High blood pressure was associated with decreased diversity (Shannon: p−val = 0.08730). Anxiety was associated with increased diversity (Tail: p-val = 0.02655; Shannon: p-val = 0.05952). Number of pets was associated with less diversity (Shannon: p-val = 0.05164).

*Greater inter-sample distances between subjects were associated with health and smoking history.* The PLCS saliva microbiota PERMANOVA analysis identified associations with days into early pandemic (p−val = 0.00030), health (p−val = 0.00030), smoking history (p−val = 0.00278), and less significantly with COVID-19 worries (p−val = 0.05685) and the number of pets (p−val = 0.04555).

*Differences in taxonomic abundances were associated with health, high blood pressure, diabetes, COVID-19 worries and asthma.* The PLCS saliva microbiota analysis using taxonomic abundances identified several associations that could support the underlying differences in diversity and distancing. Days into the early pandemic was found to be negatively associated with *Streptococcus* (p−val < 0.00001), but positively associated with *Bergeyella* (p−val = 0.00063), *Capnocytophaga* (p−val = 0.00165), and *Oribacterium* (p−val = 0.00912). Health was positively associated with *Neisseria* (p−val = 0.00007), *Alloprevotella* (p−val = 0.00613), and *Veillonellaceae*_*uncl* (p−val = 0.00719). High blood pressure was negatively associated with *Capnocytophaga* (p−val = 0.00039), *Fusobacterium* (p−val = 0.00374) and *Bergeyella* (p−val = 0.00986). Diabetes was associated with increased *Veillonella* (p−val = 0.00294). COVID-19 worries were negatively associated with *Lactobacillus* (p−val = 0.00501). Asthma was positively associated with *Yersinia* (p−val = 0.00557).

### Comparison of changes in stool and saliva microbiota profiles between pre-pandemic and early pandemic samples revealed stool microbiota communities were more stable

**Figure 3:**
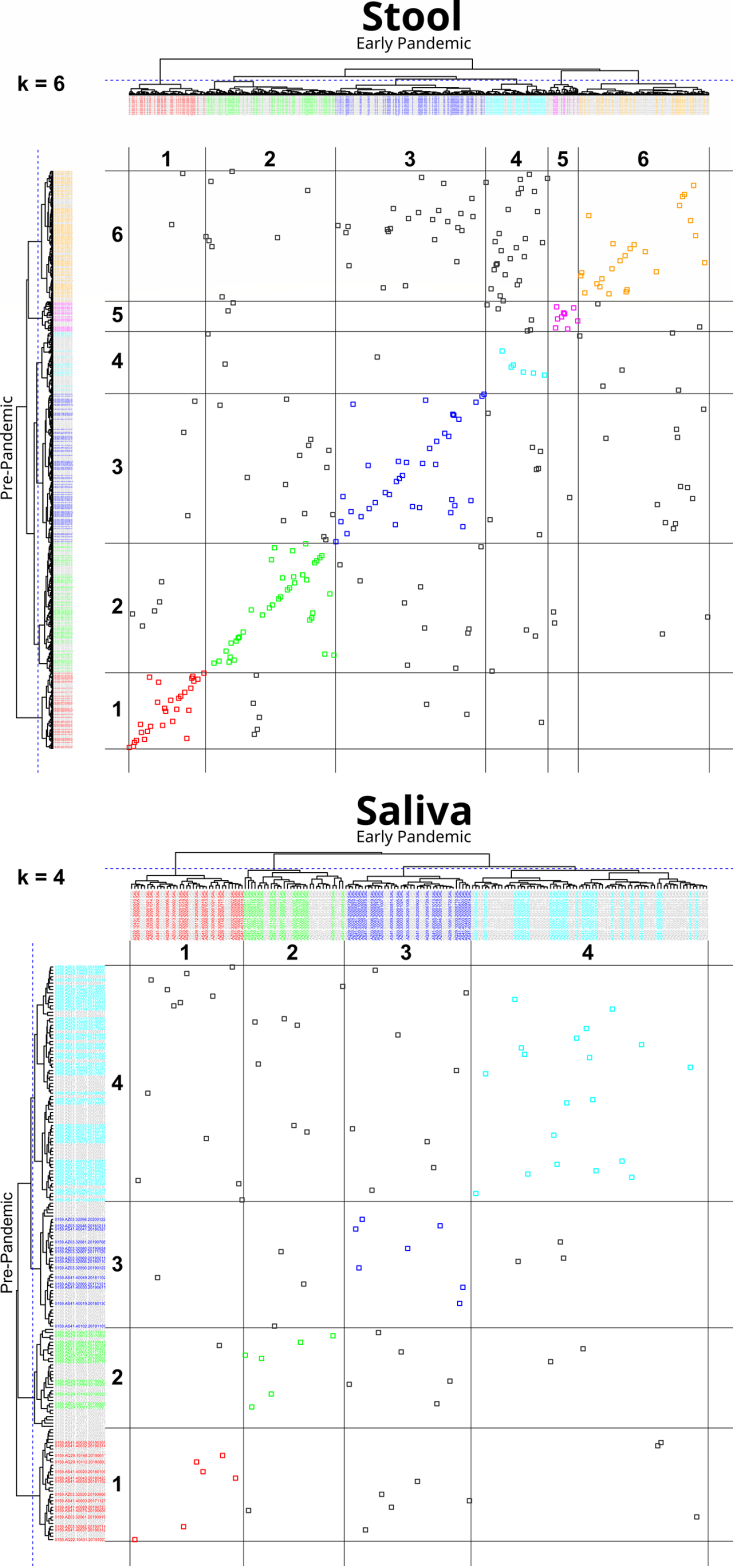
Cluster transition plot for Stool at k=6 and Saliva at k=4. Cluster transition plots provide a visualization of the degree to which an early pandemic sample’s composition has changed relative to its pre-pandemic composition to warrant a change in its cluster membership. Hierarchical clustering and tree cutting (to form discrete clusters) is inherently an iterative process. In this figure, only one slice at *k* = 6, for stool, and *k* = 4, for saliva (labeled on the top left of each plot), were selected for illustrative purposes, although cuts *k* from 2 to 7 were also calculated. The dendrogram from hierarchically clustering of pre- and early pandemic samples are drawn on the top and left margins. The left margin dendrogram have pre-pandemic samples colored by their cluster identifier, while early pandemic samples are colored grey. Similarly, but complementarily, the top margin dendrogram has early pandemic samples colored by cluster identifier, but pre-pandemic samples are colored grey. In the field of the plot, each point represents the intersection of pre- and early pandemic samples. If both pre- and early pandemic samples are in the same cluster, then they are colored by their cluster identity, otherwise they are colored grey. Gridlines are drawn in the field to help identify cluster boundaries. When pre- to early pandemic samples have changed less in their composition, their points will be colored and lie across a diagonal from bottom-left to top-right. Examples of noteworthy observations from the stool transition plot includes the number of pre-pandemic cluster 6 (*Bacteroides* and *Escherichia Shigella*) members that have moved into cluster 4 (*Bacteroides*, *Faecalibacterium*) early pandemic, or that none of the pre-pandemic members of cluster 1 (*Prevotella*, *Prevotellaceae*, and *Lactobacillus*) have moved into cluster 6. Comparing the stool and saliva cluster transition plots provides a visualization of the stronger coherence of early pandemic samples to their pre-pandemic counterparts in stool.

**Figure 4:**
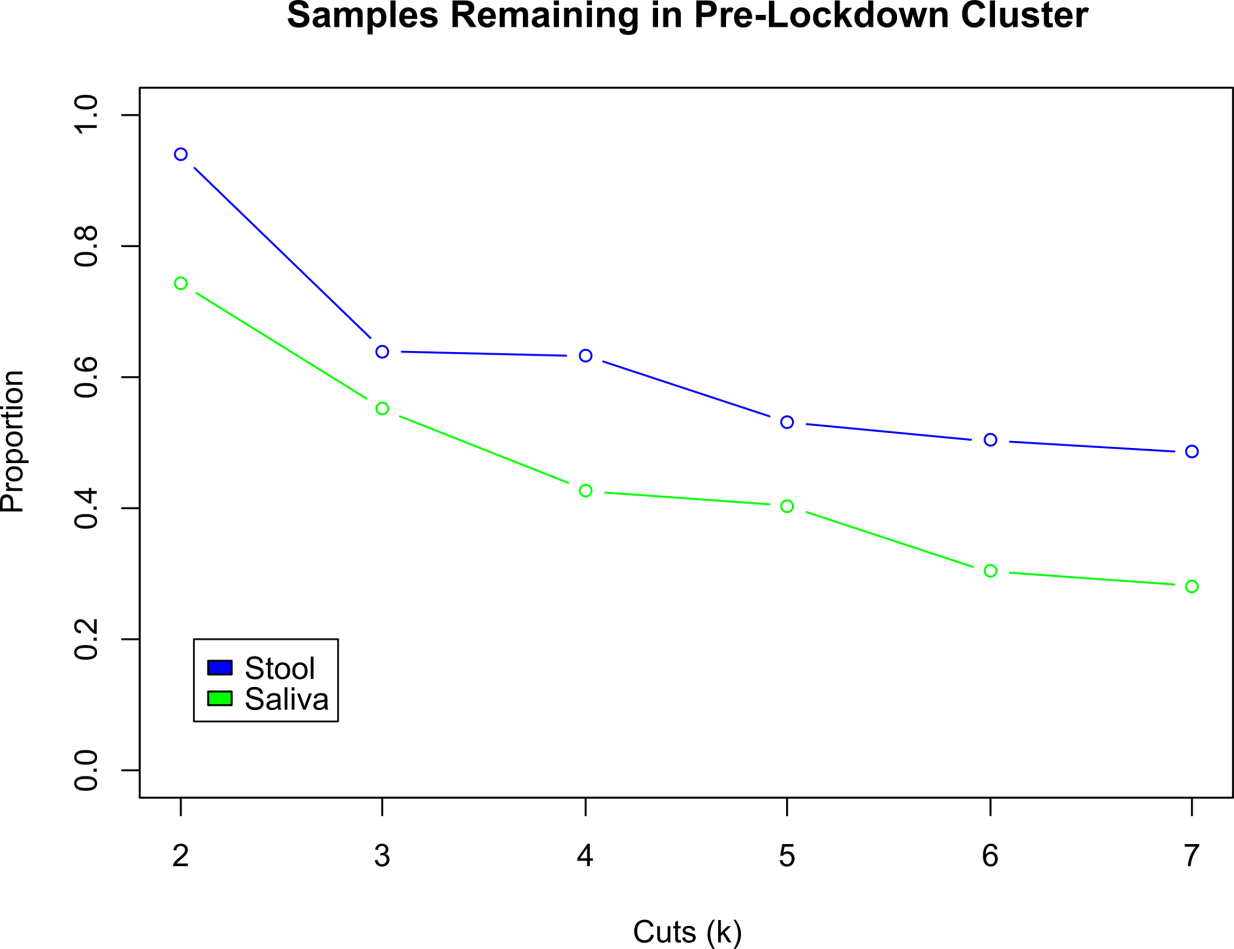
Comparison of Proportion of Subjects Changing Clusters between Stool and Saliva. These two curves illustrate the change in the proportion of early pandemic samples that remain in the same cluster as their pre-pandemic sample, for stool (blue) and saliva (green) samples. As the hierarchically clustered samples are cut from *k* = 2 to 7 clusters, the cluster sizes decrease and become more exclusive. Thus, any two samples that are in the same cluster when *k* = 7 are more similar to each other, than when *k* was smaller, e.g., 2. Across all cuts *k*, the early pandemic stool samples tend to be consistently closer to their pre-pandemic mates, than the saliva samples. At *k* = 2, the proportion of pre- and early pandemic stool samples that in the same cluster are 93.8%, compared to 74.2% in saliva. At *k* = 7, 48.6% of stool vs. 28.1% of saliva pre- and early pandemic samples are collocated in the same cluster.

An analysis of the cluster membership stability was performed separately for stool (n=288) and saliva (n=89) sample types. For each sample type, pre- and early pandemic samples were hierarchically clustered together. The resultant tree was then iteratively cut from *k* = 2 to 7 clusters and resultant memberships were evaluated. When both pre- and early pandemic samples were in the same cluster, for a specific *k*, then the subjects’ early pandemic samples were considered to have “remained” in the same cluster as the pre-pandemic sample. **Figure 3**, “Cluster Transition Scatter Plot” illustrates the cluster member relationships between pre- and early pandemic samples. The proportion of samples that remained in the sample cluster is plotted across the cuts (*k*) in **Figure 4**, “Samples Remaining in Pre-pandemic Cluster”. At *k* = 2, 93.8% of early pandemic stool samples were clustered with their pre-pandemic sample, while only 74.2% of saliva samples shared their pre-pandemic cluster. At *k* = 7, 48.6% and 28.1% of stool and saliva samples, respectively, were had their pre-pandemic and early pandemic samples clustered together. Early pandemic stool samples were consistently closer to their pre-pandemic samples than saliva samples.

Please refer to **Supplemental Table 2**, “Associations with Stool and Saliva Samples” for a complete table of coefficients and p-values for all models reported in this Result section.

## Discussion

Although effects of COVID-19 pandemic changes in human social behaviors and hygiene patterns on human microbiota and their potential interactions with the host have been postulated to include loss of diversity, they remain largely understudied. An important outcome of our study design was the ability to examine matched stool and oral sample pairs from the same individual taken from the pre-pandemic to early pandemic time points (PEPP model). Here we examined dynamic changes in alpha (within sample) diversity, and compositional changes using both measures of inter-sample distances (beta diversity) and relative taxonomic abundance. We related these diversity, distance, and abundance measures to participant questionnaire responses to determine associations with the microbiota that may have been potentiated by factors related to pandemic minimization strategies or implicit subject habits. We also examined ecological stability through pre-pandemic to early pandemic cluster transition (PEPCT) analysis. Finally, cross-sectional analyses of early pandemic (EPCS model) microbiota profiles from stool and saliva were examined to elucidate associations with health and lifestyle behavior providing a “snapshot” of these relationships at a time of heightened pandemic awareness and for the identification of study variables that may be proxies of other pre-pandemic behaviors or other lifestyle characteristics not directly measured in this study.

Early during the pandemic (2020), it was quickly established that individuals with certain comorbidities, such as hypertension or diabetes mellitus (Sanyaolu et al., 2020; Zhang et al., 2020), were at a greater risk for COVID-19 complications. As a result, individuals in our cohort (of which over half reported at least one underlying comorbidity) may have followed the recommended precautionary guidelines more strictly. Therefore, effects of the viral transmission strategies may have resulted in more substantial lifestyle changes in these individuals relative to the general population. The analyses of questionnaire responses did not identify a significant correlation between social distancing and work-from-home strategies with COVID-19 worries, as the ability to social distance may have depended more on socio-economic conditions (Garnier et al., 2021) rather than personal choice. From questionnaire responses, social distancing was negatively correlated with age, but positively correlated with asthma and COVID-19 worries.

Although both saliva and stool taxonomic profiles from matched pairs trended towards decreased (alpha) diversity, overall, from the pre-pandemic to early pandemic time points (PEPP) model, the effect was not statistically significant. In part, this finding could be due to the relatively early pandemic sampling dates. Therefore, the associations identified in this study may be limited to those factors with acute effects. In the paired analyses, the median days into the early pandemic were 162 days for stool and 172.5 days for saliva. Nonetheless, this is an interesting finding given that our cohort is older and over half of participants reported at least one comorbidity, as loss of microbiota diversity is often reported to be associated with increasing age and chronic disease (Sun et al., 2021; Ceballos et al., 2021).

Applying the pre-pandemic to early pandemic time points (PEPP) model to stool microbiota, changes in diversity were associated with changes in BMI, smoking history, and pet ownership. From our questionnaire, smoking history was reported, however changes in smoking habits from the pre-pandemic to early pandemic time periods could not be determined. Therefore, it is not clear why smoking history would decrease diversity during the early pandemic unless smoking frequency increased for this group, or if it was a proxy for another behavior. In a separate study, it was reported that during the “lockdown” phase, alcohol consumption increased, but more subjects tried to quit smoking (Jackson et al., 2021). Overall, if we assume that decreased social contact and increased hygiene measures (e.g., hand washing) can decrease diversity, then our study suggests that other factors (e.g., household pets) and those implied but not directly measured (e.g., diet) may offset these potential losses in diversity. It has also been recognized that humans can share microorganisms through social interactions, cohabitation, and exchanges with both the natural and built environments (Tong et al., 2021; Peimbert and Alcaraz, 2022). Previous reports have also identified microbiota associations with pet ownership were also found in conjunction with stool studies (Kates et al., 2020) and our study suggests that pets may be an important reservoir of microbes in humans, a relationship that may be heightened in periods of decreased social contact.

In addition to examining diversity, the pre-pandemic to early pandemic time points (PEPP) model for stool microbiota profiles also examined changes to composition as measured by paired sample distances (beta diversity) or by specific ALR-transformed taxonomic abundances. The analysis of stool microbiota paired distances found associations with pre-pandemic BMI, health, diabetes, immune system pathology, and number of cohabitants. Multiple changes in specific taxonomic abundances were associated with immune system disease, asthma, pre-pandemic BMI and changes in BMI, diabetes, depression, and education level. These results are consistent with previous findings such as a 2018 study by Rothschild and colleagues (Rothschild et al., 2018) which determined that genetic ancestry or individual polymorphic variants in families were minor contributors to gut microbiome composition (<2%), in contrast to more than 20% of the variance in microbiome diversity attributed to shared environmental, diet and lifestyle factors.

The microorganisms identified by paired sample distances or by specific ALR-transformed taxonomic abundances using the pre-pandemic to early pandemic time points (PEPP) model for stool microbiota profiles were polymicrobial in nature, however the associations almost exclusively consist of genera assigned to the dominant phyla found in the human gut, Bacteroidetes and Firmicutes. This finding suggests that this diverse set of organisms is likely to be involved in multiple human gut metabolic processes. These are likely to include the production of short chain fatty acids (Silva et al., 2020) and secondary bile acids which have been implicated in neuro-immunoendocrine regulation affecting both physical and mental health status (Romaní-Pérez et al., 2021) as well as other metabolic processes that warrant future investigation.

The pre-pandemic to early pandemic time points (PEPP) model used to examine saliva microbiota profiles, identified contrasting associations. Social distancing led to increased changes in composition, while COVID-19 worries were associated with a decrease in compositional distance, between samples from the same individual. The apparent opposition of these associations exemplifies the complexity of these host behaviors in host-microbiota interactions. While many studies have focused on the role of the microbiome in the gut-brain axis, findings in the current study suggest that saliva microbiota may in part be important contributors to, or markers of anxiety, stress, and general mental health. For example, it has been demonstrated that chronic psychological distress can depress diurnal secretion levels of salivary glucocorticoid and catecholamines (Miller et al., 2007) as well as alpha-amylase (Nater et al., 2007). Glucocorticoids, as corticosteroids, are involved in carbohydrate, protein, and fat metabolism and exhibit anti-inflammatory activity. As neurotransmitters, catecholamines, have been shown to moderate gut microorganisms (Huang et al., 2015) and may perform similar roles within the oral cavity.

Overall, paired analyses generally found fewer significant associations with saliva relative to stool microbiota. This result may in part be attributable to the smaller sample size available for the saliva analysis. However, biologically this finding may indicate that the collective effects of the variables measured within the timeframe of this study were less influential or acted more in opposition to one another in saliva microbiota relative to their stool counterparts. In addition, microbiota recovered from saliva represent an amalgam of habitats in the oral cavity, and the oral cavity has direct contact with the external environment, all factors that can result in greater sample variability.

The pre-pandemic to early pandemic cluster transition (PEPCT) analysis identified that overall, from the same subject, the microbiota profiles from both stool and saliva, from an ecological standpoint (Relman, 2012), were largely stable from pre-pandemic to early pandemic time periods with stool more stable relative to saliva. In the stool samples, departers (samples from individuals that left their pre-pandemic cluster assignment) were associated with sex (female) and COVID-19 worries. Most arrivers (samples from individuals that changed to new early pandemic cluster assignments) were associated with asthma and cancer and social distancing. These associations further support the finding that perturbations to the stool microbiota from the pre-pandemic to early pandemic period were associated with individuals reporting more issues with physical health. The association with COVID-19 worries however, may be an indicator of possible changes with mental health status or alternatively, it may be a proxy for other behaviors not measured directly in this study.

Further the pre-pandemic to early pandemic cluster transition (PEPCT) analysis provided an opportunity to identify “local shifts” or changes in microbiota composition from a subset of the cohort in relation to study variables. For instance, increased cancer diagnoses were associated with individuals that departed multiple pre-pandemic clusters but arrived in one early pandemic cluster for which cluster influencing bacteria include *Akkermansia* and *Escherichia-Shigella*. Consistent with these findings, *Escherichia* has been associated with promotion of colorectal and other cancers (Dalmasso et al., 2014), while *Akkermansia* has been linked to the potentiation of anti-CTLA-4 and anti-PD-1 immunotherapy (Miller and Carson, 2020). More recently, both microorganisms (Jayachandran et al., 2020) were found to be increased in abundance in individuals with stable non-small cell lung cancer while undergoing immunotherapy (He et al., 2021).

In a contrasting example, the pre-pandemic to early pandemic cluster transition (PEPCT) analysis was able to provide insights into specific taxa related to changes in COVID-19 worries Here the cluster influencers changed from a diverse pre-pandemic set of bacteria including *Akkermansia*, *Oscillospiraceae UCG_002*, *Bacteroides*, *Alistipes* and *Prevotella*, to a reduced set of cluster influencers consisting of *Bacteroides*, *Faecalibacterium* and *Agathobacter*. *Bacteroides* are well-known for their metabolic complexity and roles in many important metabolic activities in the human colon including a prodigious capacity to catabolize complex host and diet derived carbohydrates, as well as production of propionate, use of proteins and other nitrogenous compounds, and transformation of bile acids and other steroids (Zafar and Saier, 2021). As such, *Bacteroides* often support many interspecies cross-feeding interactions including other short chain fatty acid producers such as *Faecalibacterium* and *Agathobacter* (Rodriguez-Castaño et al., 2019). *Faecalibacterium* (Li et al., 2008) is an important producer of butyrate while *Agathobacter* fermentation products include butyrate, acetate, hydrogen, and lactate (Rosero et al., 2016). The functional significance of these microbiota compositional patterns in relation to increased COVID-19 related worries cannot ultimately be determined in this study and they could also reflect other behaviors such as pandemic-related dietary changes. However, this shift in microbiota may in part result in changes in the composition and concentration of the gut short chain fatty acid pool and other microbially-mediated metabolites consistent with results from the paired differences analyses (PEPP models). Among other biological functions, short chain fatty acids have been identified as a critical mechanism of gut-brain communication and may be relevant to the increased psychological distress reflected in greater worries about the COVID-19 pandemic (Ortega et al., 2022).

When applying the cross-sectional analyses of early pandemic (EPCS) model to microbiota profiles, contrasting associations were determined both between sample types (stool and oral) and with the previously discussed pre-pandemic to early pandemic time points (PEPP) model from both stool and oral samples. Relationships with stool microbiota largely corroborated previously identified microbiota associations with age (Yatsunenko et al., 2012; de la Cuesta-Zuluaga et al., 2019), anxiety (Foster and McVey Neufeld, 2013), health, exercise (Clauss et al., 2021), and immune system disease in studies undertaken prior to the COVID-19 pandemic. According to inter-sample distance measures, lifestyle changes, as represented by both social distancing and working from home, had a significant effect on the microbiota composition as a whole. Interestingly, these effects were not detected in the paired analyses of pre-pandemic to early pandemic time points. This finding may suggest that these associations instead serve as proxies for other pre-pandemic behavioral or other lifestyle characteristics that could be identified by their subsequent acceptance of advised changes in social distancing or work-from-home patterns during the early pandemic. Regardless of analytical method, changes in stool microbiota diversity and composition from the cross-sectional examinations were consistently related to age and more immune system disturbances. In contrast, cross-sectional analyses of early pandemic microbiota profiles from saliva (EPCS) model, identified fewer significant associations with diversity and a different set of variables related to compositional changes offering novel insights into to the effects of lifestyle and behavioral perturbations on the microbiota with influences from smoking history, COVID-19 related worries and the number of pets per household.

Strengths of our study include the relatively large number of subjects with matching pre- and early pandemic samples for both stool and saliva. The existence of an ongoing microbiome biospecimen collection and surveillance system, allowed us to rapidly integrate a COVID-19 study specific design, both quickly and practically, through sample self-collection and remotely conducted questionnaires. Analogous to long-term ecological monitoring of the environment (Vergin et al., 2013; Karl and Church, 2014), our findings argue for the importance of long-term surveillance of the human microbiome to improve the ability to monitor future potential population-wide perturbations. Further, the collection of subject covariates (e.g., BMI, age, sex, smoking, etc.) that were included into our models and are crucial to control for in microbiome studies, improved the confidence of the associations made with the questionnaire responses. While self-reported responses to questionnaires can have limitations compared to objective biomarkers (Boparai et al., 2018), we confirmed that measures of health status were consistent with clinical records.

We recognize that the study has several limitations. While the use of 16S rRNA gene sequencing, as a means to estimate the taxonomic composition of each sample can be conducted relatively quickly and with use of fewer resources, it does not measure biological function directly and therefore is limited in its ability to identify functional interactions with the host. Additional assays to elucidate potential, latent, and active metabolic process, through additional multi-omics approaches such as metagenomics, metatranscriptomics, metabolomics and personal genomic information, would provide a means to test more narrowly proposed hypotheses governing the underlying the associations determined in this study. In addition, given the rapidly changing nature of the pandemic, there may have been subsequent changes in the microbiome that we did not capture. In future works of this nature, the questionnaire could be refined by including multiple alternatively worded redundant questions that can be later combined for robustness, and by excluding some questions that may have ambiguous or unnecessary distinctions towards hypothetical physiological outcomes. We also lacked data on diet and other factors that might have influenced the microbiome.

In conclusion, our study examined changes in stool and saliva microbiota diversity and composition that may be attributable to social and lifestyle behavior mitigations from pre-COVID-19 to early pandemic time points in individuals who were not infected with SARS-CoV-2. While there was a trend towards a decrease in stool and saliva microbiota diversity, this change was not significant between pre-pandemic and early pandemic periods. Collectively, our analyses support the notion of relative ecological stability in stool and saliva microbiota taxonomic profiles (with higher stability found in stool) from the pre-pandemic to early pandemic periods. Greater changes in microbiota diversity and taxonomic profiles were associated with more questionnaire reported health issues including immune system disturbances, asthma, and cancer, or with greater worries related to the COVID-19 pandemic. Therefore, managing underlying comorbidities and psychological distress such as worries about the pandemic may be important for maintaining beneficial host-microbiome interactions. Our study highlights the importance of longitudinal sampling of large observational cohorts as a valuable tool to examine the status of the microbiome over time in response to pandemics and changes in public health measures.

## Data availability statement

The original contributions presented in the study are publicly available in NCBI using accession number PRJNA847970.

## Ethics statement

The studies involving human participants were reviewed and approved by University of Pittsburgh Institutional Review Board under protocol number CR19030104-017. The patients/participants provided their written informed consent to participate in this study.

## Author contributions

KL analyzed data and drafted the manuscript. CK and VP completed questionnaires. HG conducted specimen processing. AP and AF performed laboratory processing to generate data. AF and PS conducted data QC and management. AM conceived the study, provided specimen management, clinical review and edited the manuscript. BM generated and analyzed data and drafted and edited the manuscript. All authors contributed to the article and approved the submitted version.

## Funding

This work was funded by the UPMC Immune Transplant and Therapy Center (ITTC).

## Conflict of interest

The authors declare that the research was conducted in the absence of any commercial or financial relationships that could be construed as a potential conflict of interest.

## Publisher’s note

All claims expressed in this article are solely those of the authors and do not necessarily represent those of their affiliated organizations, or those of the publisher, the editors and the reviewers. Any product that may be evaluated in this article, or claim that may be made by its manufacturer, is not guaranteed or endorsed by the publisher.
