# Supplementary material for: Gut and oral microbiota associations with viral mitigation behaviors during the COVID-19 pandemic
Source: Front Cell Infect Microbiol. 2022 Sep 9;12:966361. doi: 10.3389/fcimb.2022.966361 (PMC9500509; doi:10.3389/fcimb.2022.966361)
Supplement: Supplementary file 1 [file Presentation_1.pdf]

Supplemental

Methods

Cluster Influencers Analysis

The purpose of the cluster influencers analysis is to identify which taxa are responsible for a cluster's separation from its peer clusters. Clustering depends on the distance metric (e.g., Euclidean, Manhattan, Morisita-Horn, Jaccard, etc.) used to compare samples and the clustering algorithm (e.g., Ward's minimum variance, complete-linkage, etc.) used to join (or split) members together (or apart) into clusters. For compositional data, taxonomic categorical counts for each sample are first normalized (to sum to 1) to produce relative abundance profiles, then a distance metric is calculated pairwise between these profiles. Since the same dataset may yield different clusters depending on metric and clustering algorithm, the separation between two clusters cannot be simply attributed to differences in their taxonomic abundance, as this was not the criteria used that separated them.

To identify the taxonomic contributors to cluster separation, an analysis of variance (ANOVA) approach is taken by using the R^2 statistic between two clusters to measure cluster separation. Here we define R^2 as the sum of squares between (SSB) / sum of squares total (SST). To determine whether a taxon of interest contributed to the separation between two clusters, we first calculate the reduced-R^2, which is the R^2 when the taxon of interest is removed from all samples, then we compare it against the full-R^2 which includes all taxa. If the log-ratio of the reduced-R^2/full-R^2 is negative, then that taxon contributed to the two clusters separating from each other. If the log-ratio is positive, then that taxon contributed "noise" to the two clusters and can be ignored. This is done for all taxa of interest, usually the top 15-35 taxa in abundance. Taxa that contributed to cluster separation are called "Cluster Differentiators". Taxa that are consistently cluster differentiator against other clusters, are called "Cluster Unifiers". A set of cluster unifiers is considered the defining characteristic of that cluster, or a microbiome "type" or configuration.

Since hierarchical clustering was used to generate our clusters, we also use an iterative tree cutting algorithm to generate cluster cuts from cuts k = 2 to 10, i.e. (k-1) groups of clusters, with cluster identifiers in each grouping labeled cl = 1 to k. Cluster unifiers are calculated for each cut and summarized in a hierarchically structured table. See Supplemental Figure 5 and Figure 6 for the cluster unifiers for stool and saliva, respectively.

Cluster Transition Analyses

The goal of cluster transition analysis is to understand how a cohort's microbiota has changed between their pre- and early pandemic sample, given their pre-pandemic microbiota composition and factors, such as demographics and questionnaire responses. The assumption that is made when modeling with clustering is that a cluster represents a particular state or configuration of the microbiota from a sample that cannot be meaningfully reduced further. While there may be an infinite number of possible compositions that can be brought together in a cluster, each variation is not considered consequential when considering that cluster as whole. In other words, the members of the cluster may be treated homogenously if they clustered together, and should be considered different in state of configuration, if they cluster separately.

To identify the possible microbiota states that may exist in a cohort, both the pre- and early pandemic samples were clustered together. Pairwise sample distancing was performed with the Manhattan distance metric, followed by hierarchical clustering with Ward's minimum variance criterion. The tree resultant from hierarchical clustering was cut iteratively from k = 2 to 7. At each cut, a *k* x *k* contingency table was computed with the rows and the columns containing the pre- and early pandemic cluster identifiers, respectively. For example, at k = 2, a contingency table would be a 2 x 2 matrix. Please examine Figure 3, "Cluster Transition", for examples of stool and saliva sample "contingency tables" drawn as a scatter plot, cut at k = 6 and k = 4, respectively.

When a sample's pre- and early pandemic sample are in the same cluster, they are considered to have not changed clusters, i.e., state or configuration. From these initial data structures, conditional and joint probabilities may be calculated to quantify whether some pre-pandemic clusters have a stronger probability of transitioning towards one early pandemic cluster versus another. To incorporate questionnaire responses and demographic data, two models were fit: "Departers" and "Arrivers". In the "Departers" model, the members of each pre-pandemic cluster were split into two groups, the "remainers" and the "departers". The pre- and early pandemic samples of the remainers share the sample cluster id. The departers pre- and early pandemic belong in different clusters. For each pre-pandemic cluster, a logistic regression was fit with the departers/remainers as a response to subject demographics and questionnaire responses. See Supplemental Figure 4. "Cluster Transition Departers". In contrast, for the "Arrivers" model, the members of each early pandemic cluster were examined. Remainers were subjects that had both pre- and early pandemic samples in the same cluster, whereas the "arrivers" had a sample from a different pre-pandemic cluster. Logistic regression was then computed with the arrivers/remainers as a response to subject demographics, questionnaire responses, and pre-pandemic cluster id. Thus, the "Arrivers" analysis is conditional on the subjects' pre-pandemic state. See Supplemental Figure 3. "Cluster Transition Arrivers".

Since the association between model predictors (demographics and questionnaire responses) depends on the cut *k*, the logistic regressions are calculated across all the clusters (identified 1 to k) generated at each cut. P-values are accumulated for each predictor across all the cuts, and the cut with the most significant p-value is considered the best. If variable-to-cluster associations from multiple cuts of k are equally significant, then the cut k with the lowest value is considered the best association/cut combination, by Occam's razor.

P-values

P-values have not been adjusted for multiple testing. Adjustments for multiple testing assume every test is independent. Our analyses on diversity, distance, and abundance frequently overlap in their associations as the same taxa are contributing to the three different analyses, thus correcting for each test would over penalize the significance of each calculated association. With respect to factors included in the model, some demographic variables may be considered nuisance variables, such as age, ethnicity, sex, and BMI (for cross-sectional analyses), since these may not be of direct interest, but necessary to control for. Each of the taxa considered in the abundance analyses, 25, should be considered separate tests, but the reader should be more discerning

towards accepting statistically significant associations with taxa with lower abundance. Excluding demographic variables, the number of questionnaire responses included in the models were 21. A summary of noteworthy associations that were included in the manuscript have been included into Supplemental Table 1. “Associations Comparisons”.

R Analytical Code

The R code used for the “Cluster Influencers Analysis” and “Cluster Transition Analyses” has been provided on GitHub at:

https://github.com/CMM-Release/MB-COVID

The software was written to be executed from the Linux command line and is part of a larger suite of analytical tools. Experience with R programming, cluster analyses, ANOVA, and multinomial logistic regression is suggested.

Supplemental

Tables

	Stool	Saliva
Questionnaire Repondents	**588**	**588**
Early Pandemic (EP) Respondents Excluded	*(277)*	*(370)*
EP Cross-Sectional Analysis Subjects	**311**	**218**
EP Subjects Excluded	*(23)*	*(129)*
PP-EP Paired Analysis Subjects	**288**	**89**

Supplemental Table 1, “Sample Exclusions”

This table reports the sample sizes that were available for the statistical analyses of the stool and saliva samples. From the ongoing MedBio observational cohort, 588 questionnaires responses were collected. For the early pandemic (EP) cross-sectional analyses, 277 stool and 370 saliva contributing subjects were excluded for reasons including: difficulty collecting early pandemic samples during lockdown, unavailable BMI information, or 16S rRNA gene sequencing not passing quality control (QC). For the paired analyses, 23 stool and 129 saliva EP samples were excluded because they lacked a matching pre-pandemic sample. The most common reason for the exclusion of EP subjects was that although they were enrolled, they had not provided the necessary sample type prior to the lockdown date cutoff. The majority of subjects providing saliva samples, also provided stool samples.

Stool

Early Pandemic Cross-Sectional

**Diversity**	**Coefficient**	**P-value**
Immune System Disease / Tail	-1.137	0.0000
Immune System Disease / Shannon	-0.685	0.0000
Age / Tail	0.025	0.0003
Age / Shannon	0.018	0.0003
Health / Tail	0.230	0.0246
Health / Shannon	0.157	0.0282
GAD7 Anxiety / Shannon	0.134	0.0676
Pre-Pandemic Exercise / Shannon	0.279	0.0689
Pre-Pandemic Exercise / Tail	0.374	0.0886

**Distance**	**R^2**	**P-value**
Days Into Early Pandemic	0.015	0.0000
BMI	0.010	0.0016
Age	0.012	0.0004
Health	0.011	0.0005
Sex (Female)	0.019	0.0000
Immune System Disease	0.016	0.0000
Social Distancing	0.005	0.0627

**Abundance**	**Coefficient**	**P-value**
Sex (Female) / Prevotellaceae_uncl	-1.532	0.0000
Sex (Female) / Prevotella	-1.480	0.0002
Sex (Female) / Bacteroides	0.828	0.0008
Age / Alistipes	0.053	0.0000
Immune System Disease / UCG_002	-1.638	0.0000
Immune System Disease / Subdoligranulum	-1.690	0.0000
Immune System Disease / Ruminococcus	-1.538	0.0002
Immune System Disease / Lachnospiraceae_NK4A136_grp	-1.112	0.0006
Immune System Disease / Fusicatenibacter	-1.189	0.0008

Pre / Early Pandemic Paired

**Diversity**	**Coefficient**	**P-value**
dBMI / Tail	-0.229	0.0232
Smoking History / Tail	-0.781	0.0317
Number of Pets / Tail	0.709	0.0406

**Distance**	**Coefficient**	**P-value**
Days into Pre-Pandemic	0.000	0.0023
Days into Early Pandemic	0.002	0.0265
Pre-Pandemic BMI	-0.010	0.0328
Health	-0.068	0.0178
Diabetes	0.151	0.0708
Number of Cohabitants	0.080	0.0907

**Abundance**	**Coefficient**	**P-value**
Immune System Disease / Alistipes	1.298	0.0000
Immune System Disease / Lachnospiraceae_uncl	0.694	0.0010
Immune System Disease / Bacteroides	0.874	0.0011
Immune System Disease / Faecalibacterium	0.971	0.0057
Asthma / Ruminococcus	1.860	0.0008
Days into Pre-Pandemic/ Fusicatenibacter	0.002	0.0003
Days into Early Pandemic / Lachnoclostridium	0.015	0.0005
Pre-Pandemic BMI / Prevotella	0.119	0.0004
dBMI / UCG_002	-0.276	0.0004

Days into Early Pandemic / Prevotella	-0.025	0.0002	dBMI / Escherichia_Shigella	0.301	0.0045
Days into Early Pandemic / Prevotellaceae_uncl	-0.013	0.0005	dBMI / Subdoligranulum	-0.219	0.0046
BMI / Bacteroides	0.081	0.0006	Diabetes / Agathobacter	1.508	0.0099
BMI / Lachnoclostridium	0.082	0.0006	PHQ9 Depression / Lachnospiraceae_uncl	-0.378	0.0025
			Education Level / Prevotella	0.354	0.0088

**Cluster Transistion**	**Coefficient**	**P-value**
Sex (Female) / Depart cl=2, k=3	-2.751	<0.001
COVID Worries / Depart cl=2, k=6	0.442	0.0060
COVID Worries / Arrive cl=3, k=6	-0.370	<0.001
Asthma / Arrive cl=1, k=2	4.197	0.0090
Cancer / Arrive cl=2, k=5	1.630	0.0050
Social Distance / Arrive cl=1, k=2	4.197	0.0090

Saliva

**Diversity**	**Coefficient**	**P-value**	**Diversity**	**Coefficient**	**P-value**
Early Pandemic BMI / Tail	0.045	0.0150	Immune System Disease / Tail	1.261	0.0116
Early Pandemic BMI / Shannon	0.026	0.0190	Education Level / Tail	-0.344	0.0687
High Blood Pressure / Shannon	-0.197	0.0873	PHQ9 Depression / Tail	0.583	0.0719
GAD7 Anxiety / Tail	0.260	0.0266	Number of Cohabitants / Tail	-0.774	0.0973
GAD7 Anxiety / Shannon	0.133	0.0595	GAD7 Anxiety / Tail	-0.472	0.0986
Number of Pets / Shannon	-0.223	0.0516			

**Distance**	**R^2**	**P-value**	**Distance**	**Coefficient**	**P-value**
Days into Early Pandemic	0.020	0.0003	Social Distancing	0.265	0.0277
COVID Worries	0.009	0.0568	COVID Worries	-0.027	0.0754
Number of Pets	0.009	0.0456			
Health	0.021	0.0003			
Smoking History	0.016	0.0028			

**Abundance**	**Coefficient**	**P-value**	**Abundance**	**Coefficient**	**P-value**
Days into Early Pandemic / Streptococcus	-0.015	0.0000	COVID Worries / Oribacterium	0.276	0.0030

Days into Early Pandemic / Bergeyella	0.012	0.0006	COVID Worries / Campylobacter	0.190	0.0085
Days into Early Pandemic / Capnocytophaga	0.013	0.0017			
Days into Early Pandemic / Oribacterium	0.012	0.0091			
Health / Neisseria	1.026	0.0001			
Health / Alloprevotella	0.583	0.0061			
Health / Veillonellaceae_uncl	0.582	0.0072			
High Blood Pressure / Capnocytophaga	-0.898	0.0004			
High Blood Pressure / Fusobacterium	-0.705	0.0037			
High Blood Pressure / Bergeyella	-0.548	0.0099			
Diabetes / Veillonella	0.885	0.0029			
COVID Worries / Lactobacillus	-0.183	0.0050			
Asthma / Yersinia	0.773	0.0056			

Supplemental Table 2, “Associations with Stool and Saliva Samples”.

This table summarizes the significant associations found in the stool and saliva analyses organized so that the various metrics and methods may be compared. Each association includes a description, coefficient, and p-value. The left and right columns contain the associations for the “Early Pandemic Cross-Sectional” analyses and “Pre / Early Pandemic Paired” analyses, respectively. The top and bottom analyses are split into “Stool” and “Saliva” analyses, respectively. Within each sample type by analyses grouping, the sub-analyses “Diversity”, “Distance”, and “Abundance” are shown. The diversity analyses contain associations made with the Tail or Shannon diversity index. The distance analyses used the Manhattan distance. The abundance analyses used the additive-log-ratio transformed abundances. P-values are not adjusted for multiple testing, but only the most noteworthy were included (See Supplemental Methods P-values).

Supplemental

Figures

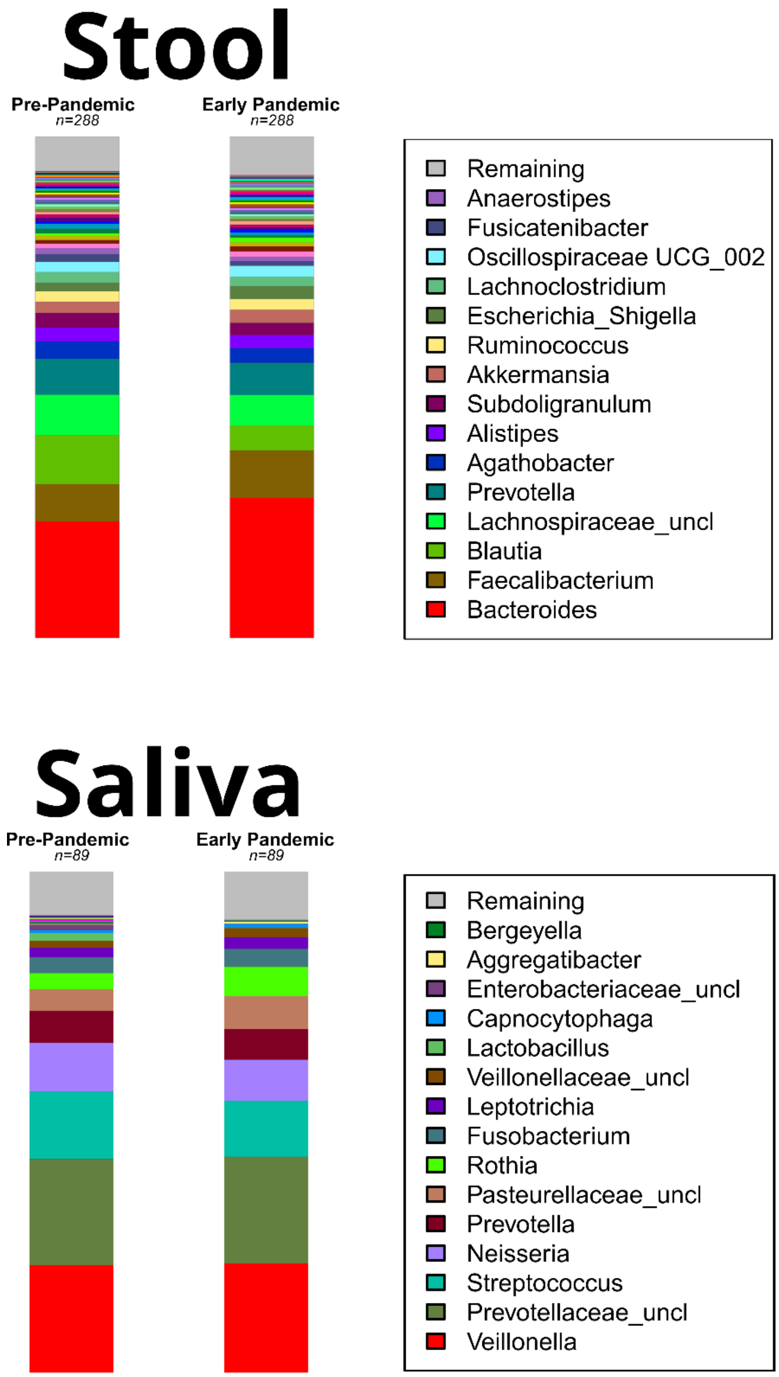

Supplemental Figure 1, “Paired Compositional Stacked Bar Plots”

The top and bottom stacked bar plots represent the average microbiota compositions for stool and saliva samples, respectively, that were analyzed for the paired analyses. The pre- and early pandemic compositions are represented on the left and right, respectively. The most abundant taxa are located on the bottom of the stacked bar plots and their corresponding color coding can be found in in the sample type specific legend on the right. The most abundant taxa in stool included *Bacteroides* and *Faecalibacterium*. The most abundant taxa in saliva included *Veillonella* and *Prevotellaceae*. Only the top 15 taxa were labelled, although additional taxa were represented. Taxa with abundances too small to represent with colors were placed into the grey “Remaining” category.

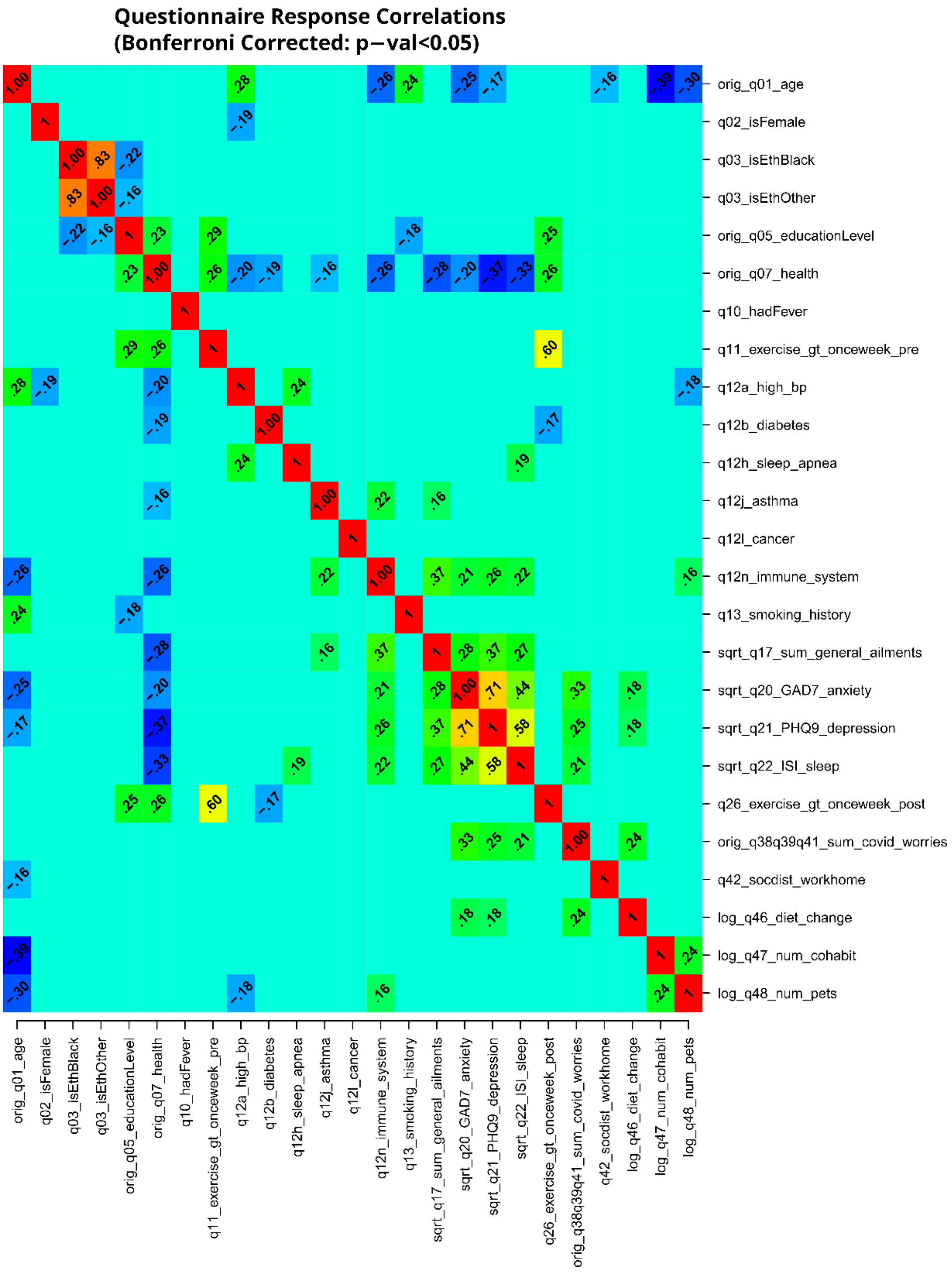

Supplemental Figure 2, “Questionnaire Response Correlations”

This heat map contains the most significant (Bonferroni correct p-values < 0.05) correlation coefficients calculated pairwise between all questionnaire responses used in the analyses. Positive correlations are colored towards red and negative correlations are colored towards blue. The number of statistical tests corrected for only included the number of cells on one side of the diagonal. Variable names include the question identifier(s) and whether they were first, logarithmically or square root transformed for normality for downstream analyses. The “orig” variable name prefix indicates that the variable’s distribution was not normal, but neither transforms could improve their normality (measured with the Shapiro-Wilk test), so their original untransformed values were retained.

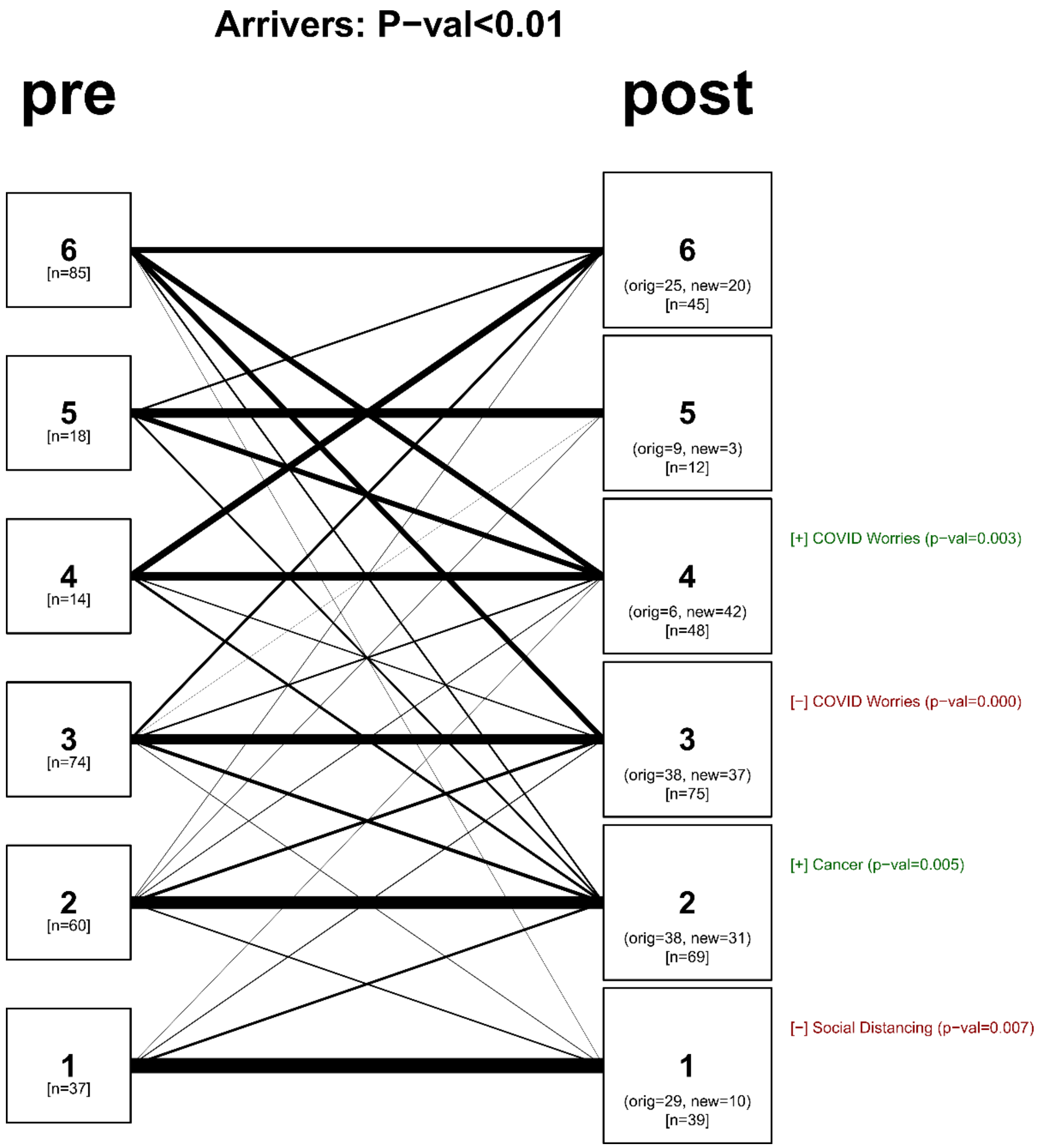

Supplemental Figure 3, "Stool Cluster Transition Departers at k = 6"

Cluster departers are subjects whose early pandemic samples do not cluster together with their pre-pandemic samples. When the hierarchical clustering was cut at k = 6, a positive association between COVID Worries (p-value = 0.006) and the departers from cl = 3 was identified. Of the original 74 subjects in cl = 3, 36 (48.6%) left (went to another cluster) and 38 (51.4%) stayed in the same cluster. Questionnaire questions were associated with departers with logistic regression. Although this figure represents k = 6, this analysis was performed across cuts k = 2 to 7. Each questionnaire response may find its associations with departers at multiple cuts of *k*, so the cut with the most significant association is reported per questionnaire response.

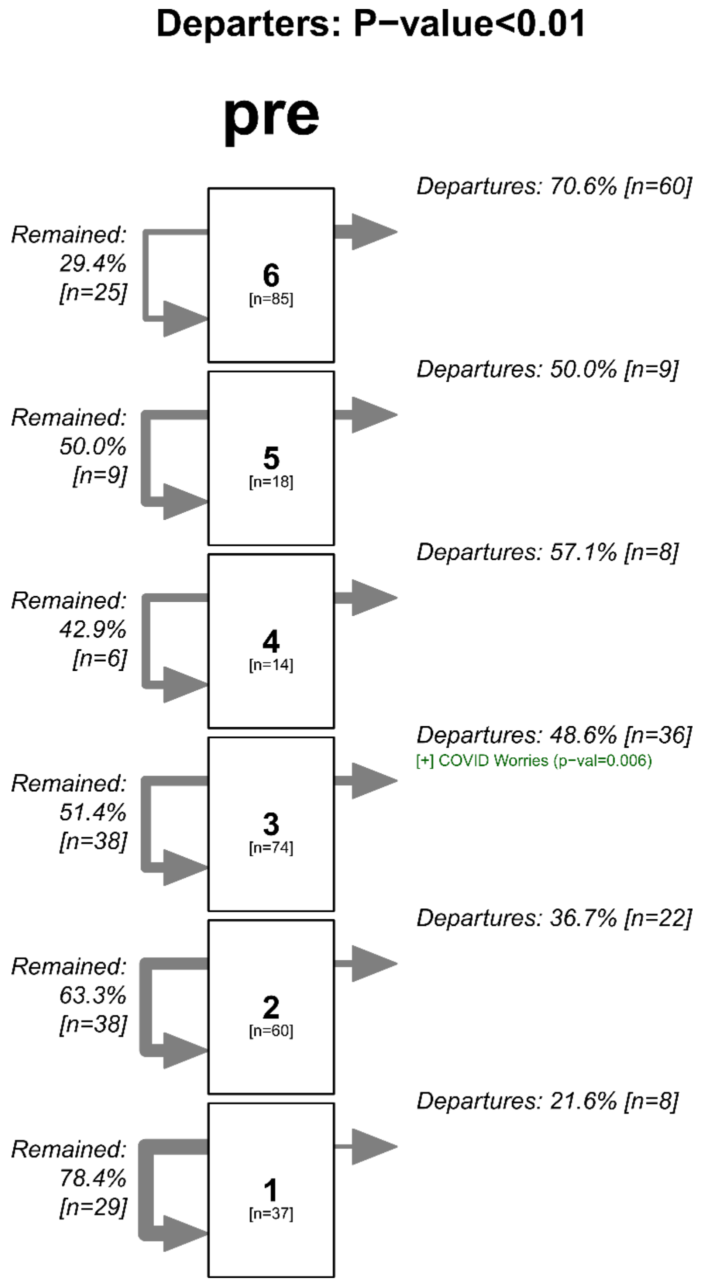

Supplemental Figure 4, “Stool Cluster Transition Arrivers at k = 6”

The cluster arriver analyses identify the factors that are associated with a subject moving into an early pandemic cluster. When the hierarchical clustering was cut at k = 6, COVID Worries was associated with more subjects transitioning into cl = 4 and fewer subjects transitioning into cl = 3. There were more subjects transitioning into cl = 2 associated with Cancer, and fewer transitioning into cl = 1 associated with Social Distancing. The cluster sizes of the pre-pandemic and early pandemic clusters are annotated in each cluster square. The line thickness connecting pre-pandemic to early pandemic clusters is proportional to the number of subjects moving between pre- and early pandemic clusters. The early pandemic clusters are annotated with cluster size as well as the composition of original and newly arriving members. Logistic regression was used to calculate associations with questionnaire responses while controlling for pre-pandemic cluster membership. Although associations with Cancer and Social distancing are show in this figure, Cancer had a stronger association at (k=5, cl = 2), and Social Distancing had a strong association at (k=10, cl=7).

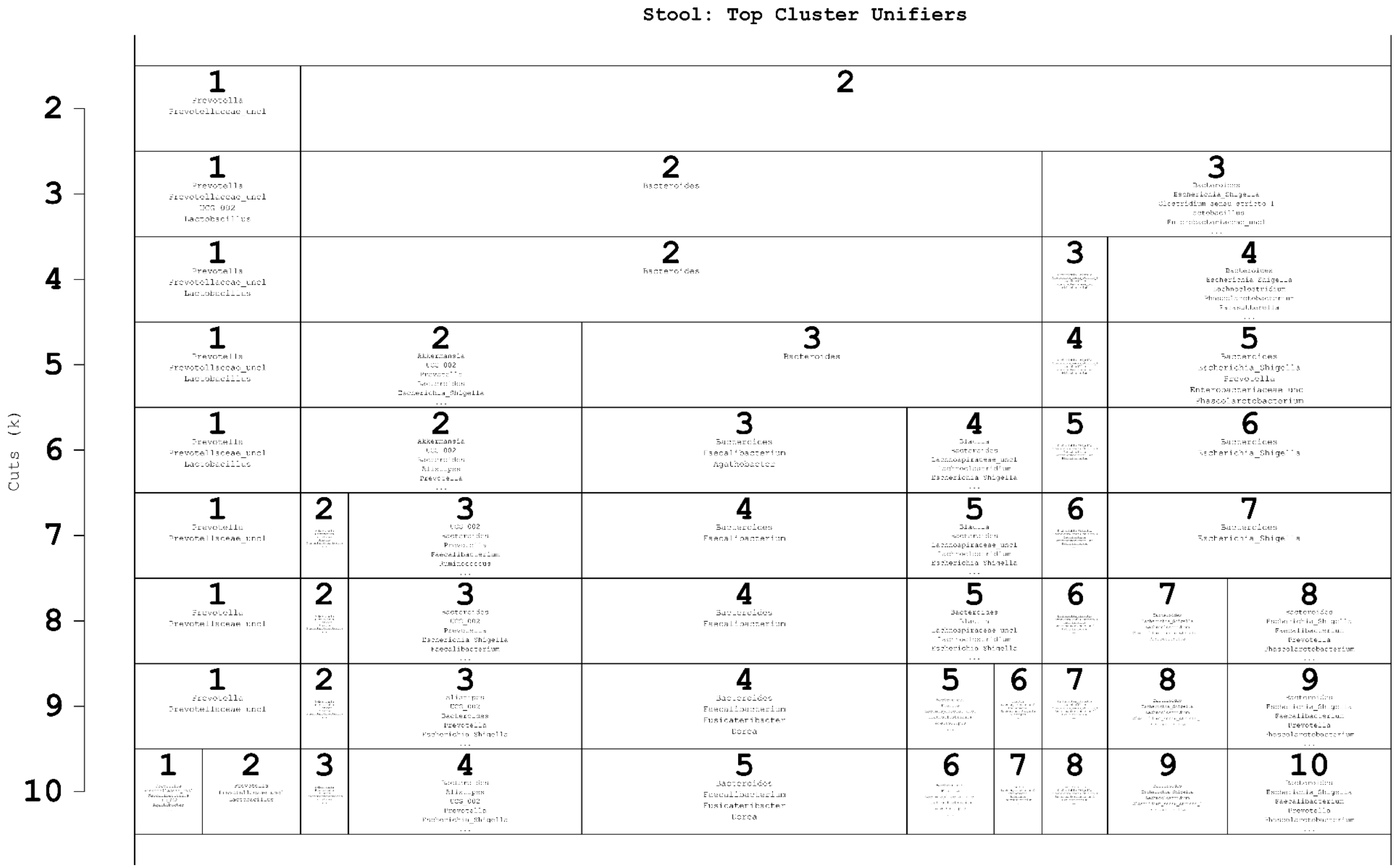

Supplemental Figure 5, “Stool Cluster Unifiers”

Cluster unifiers for the pre- and early pandemic stool samples are organized into a hierarchically structured table. Each row represents a cut from k=2 to 10, labeled on the left y-axis. Each row represents the clusters that are generated when the samples are cut, with the relative width of each cell proportional to the cluster size. The cluster identifiers, cl = 1 to k, are annotated in each cell. The taxa that unify each cluster are displayed under each cluster identifier. When there is no taxa labeled, the cluster is too heterogeneous to be described, e.g. at (k =2, cl = 2).

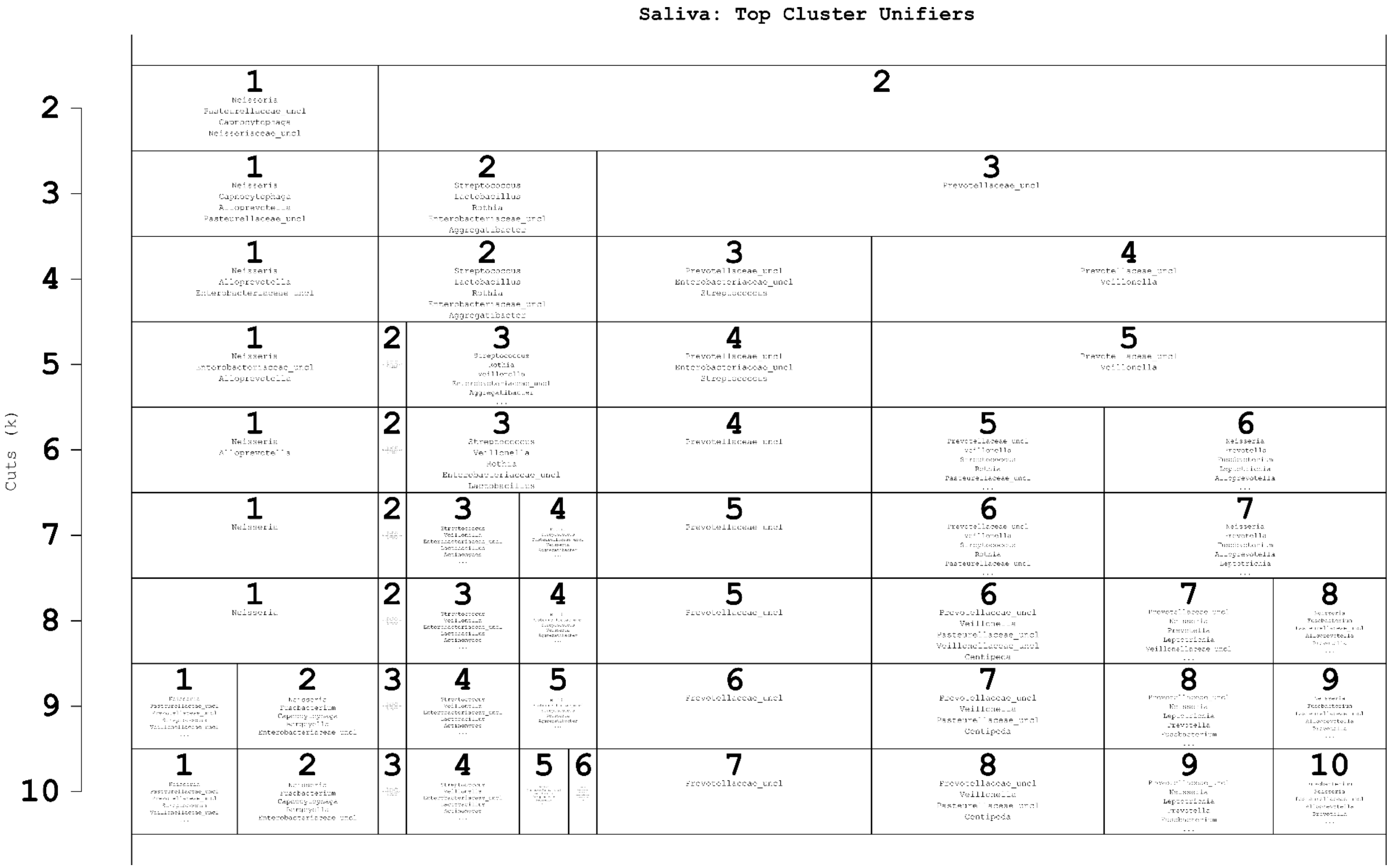

Supplemental Figure 6, “Saliva Cluster Unifiers”

Cluster unifiers for saliva are illustrated in this figure. See the figure caption for the “Stool Cluster Unifiers” for a more thorough description.

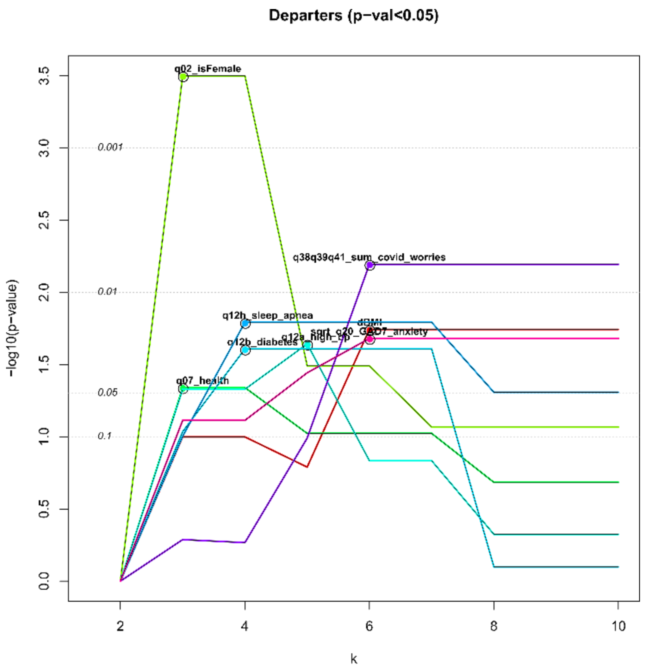

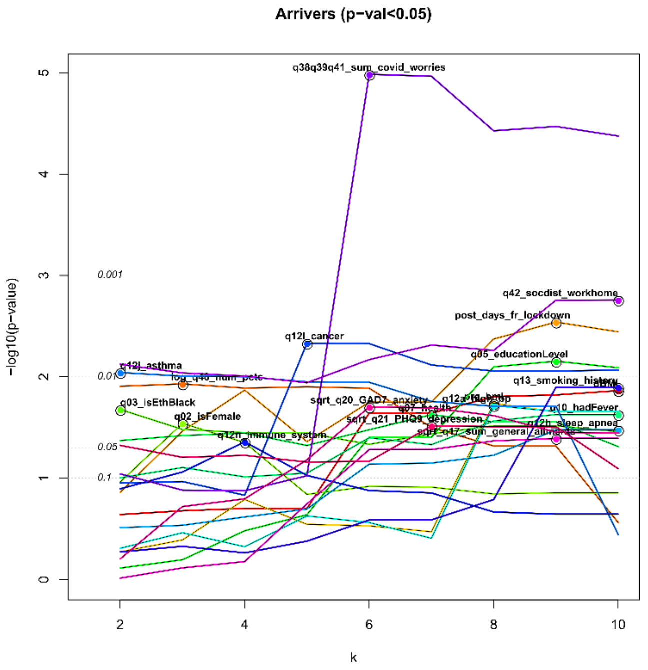

Supplemental Figure 7, “Stool Cluster Transition Associations by Cutoff”

These plots illustrate the suggested cluster k cutoff for each variable included in the cluster transition analysis. The top and bottom plots are calculated for the cluster departers and arrivers, respectively. The x-axis indicates the cluster cut *k* value, and the y-axis indicates the log(p-value) of the most significant association for each variable across the k clusters. The greater the p-value of the association between k and variable, the less homogenously the variable’s values are spread across the clusters. In the departers plot, it appears that when the hierarchical clusters are split into k = 2 clusters, one of the clusters has significantly more Females departing. In the arrivers plot, it appears that when the hierarchical clusters are split into k = 6 clusters, the COVID worries tended to come to the same cluster.
