# Supplementary material for: Gut and oral microbiota associations with viral mitigation behaviors during the COVID-19 pandemic
Source: Front Cell Infect Microbiol. 2022 Sep 9;12:966361. doi: 10.3389/fcimb.2022.966361 (PMC9500509; doi:10.3389/fcimb.2022.966361)
Supplement: Supplementary file 2 [file DataSheet_1.zip › MedBio COVID Questions.pdf]

MedBio COVID-19 Baseline Questionnaire

Study ID: ___________

DATE:

MedBio ID: ___________

ZIP code (if in the U.S.) or postal code of your primary residence:

____

1. **What is your age in years?** _________

2. **What is your biological sex?** ☐ Male ☐ Female ☐ Prefer not to say

3. **What is your racial background? (Check all that apply)**
   - ☐ Black or African American
   - ☐ White
   - ☐ Asian (including South Asian and Asian Indian)
   - ☐ Native Hawaiian or Pacific Islander
   - ☐ American Indian or Alaska Native
   - ☐ Some other race
   - ☐ Don’t know

4. **Are you of Hispanic, Latino or Spanish origin or ancestry?**
   - ☐ No
   - ☐ Yes – Mexican, Mexican American or Chicano
   - ☐ Yes – Puerto Rican
   - ☐ Yes – Cuban
   - ☐ Yes – Other
   - ☐ Don't know or Prefer not to state

5. **What is the highest level of education you have achieved?**
   - ☐ Did not graduate high school
   - ☐ High school diploma or equivalency (e.g., GED)
   - ☐ Associate degree or at least 2 years of college
   - ☐ Bachelor's degree
   - ☐ Master's degree
   - ☐ Doctorate
   - ☐ Don't know or Prefer not to state

6. **What is your marital status?**

- ☐ Married or living as married
- ☐ Widowed
- ☐ Divorced or separated
- ☐ Never married

PAST Health History

7. **In general, would you say your health is:**

- ☐ Excellent
- ☐ Very good
- ☐ Good
- ☐ Fair
- ☐ Poor

8. **Did you have a flu shot (influenza vaccine) in the past year?**

- ☐ Yes
- ☐ No
- ☐ Don’t know

9. **How many total cold or flu illnesses have you had in the past year?**

- ☐ None
- ☐ 1
- ☐ 2
- ☐ 3 or more
- ☐ Don’t Know

10. **How many total cold or flu illnesses associated with a fever (temperature > 101.3°F or 38.5°C) have you had in the past year?**

- ☐ None
- ☐ 1
- ☐ 2
- ☐ 3 or more
- ☐ Don’t Know

10b. When was the last one? __________ weeks ago

11. **On average <u>during the past year</u>, how often have you exercised more than 20 minutes (enough to breathe heavily and/or sweat)?**

- ☐ Less than once a month
- ☐ 1 – 3 Times a month
- ☐ 1 – 3 times a week
- ☐ More than 3 times

12. **Have you ever been diagnosed with any of the following?**

	Yes	No	Don't know
a. High blood pressure (except that occurred during pregnancy and did not last after pregnancy)			
b. Diabetes (not including pre-diabetes)			
c. Coronary artery disease (blockages in your heart vessels) or angina (chest pain)			
d. Heart attack (myocardial infarction)			
e. Congestive heart failure (CHF, heart failure)			
f. Stroke or TIA (transient ischemic attack or mini-stroke)			
g. Atrial fibrillation (Afib, AF)			
h. Sleep apnea (obstructive sleep apnea, OSA)			
i. COPD (emphysema, chronic bronchitis, obstructive pulmonary disease)			
j. Asthma (still requiring inhalers)			
k. Other lung disease			
l. Cancer (including leukemia or lymphoma) undergoing active treatment			
m. Pulmonary embolism or deep vein thrombosis (PE, DVT, blood clots in the lungs or legs)			
n. Disease of immune system or on drug that suppress immune system (not including HIV)			
o. Chronic HIV infection			
p. Anemia or other blood disorder (not including leukemia or lymphoma)			
q. Are you currently pregnant? (Females Only)			

Smoking History

13. **In your entire life have you smoked 100 or more cigarettes (5 packs or more)?**

☐ Yes
☐ No
☐ Don't know

13b. If yes, have you smoked cigarettes in the past 30 days?

☐ Yes
☐ No
☐ Refuse to answer

13c. On average, how many cigarettes per day have you smoked in the past 30 days? (Use 1 if less than one.) ________ cigarettes

14. **Have you smoked marijuana in the past 30 days?**

☐ Yes
☐ No
☐ Don't know
☐ Refuse to answer

15. **Have you used an electronic nicotine product in the past 30 days?**

- ☐ Yes
- ☐ No
- ☐ Don't know
- ☐ Refuse to answer

15b. If yes, what type of e-cigarette/vape product did you use?

- ☐ Nicotine
- ☐ Marijuana
- ☐ Both

15c. How many puffs from an e-cigarette do you typically take over the past 30 days? _______ puffs

Recent Health History

16. **Have you been tested for the novel coronavirus (the virus that causes COVID 19)?**

- ☐ Yes
- ☐ No
- ☐ Other, please specify: ____

16b. If yes, what was the result?

- ☐ Positive
- ☐ Negative
- ☐ I don't know yet
- ☐ Other, please specify: ____

16c. How many weeks ago were you tested? (Put 0 if tested this week.) _______ week(s)

17. **Have YOU had any of the following since March 1st (new or worse than your usual, CHECK ALL THAT APPLY):**

- ☐ Scratchy or painful sore throat
- ☐ Cough
- ☐ Shortness of breath or difficulty breathing
- ☐ Symptoms of fever or chills
- ☐ Temperature greater than 101.3°F
- ☐ Muscle aches
- ☐ General fatigue or extreme tiredness
- ☐ Diarrhea
- ☐ Nausea or vomiting
- ☐ Loss of sense of taste or smell
- ☐ None of the above

17b. If you have had any of the following symptoms, did you seek medical care for these symptoms?

- ☐ Yes
- ☐ No

18. **Have you been hospitalized (had an overnight stay in a hospital) since March 1st?**

- ☐ Yes, a UPMC hospital.
- ☐ Yes, NOT a UPMC hospital.
- ☐ No

18b. When were you discharged from the hospital (if more than one time, use most recent)?

________ / ________ / ________
MM DD YYYY

18c. What was the main reason for your most recent hospitalization (you can look at the papers you received at discharge from the hospital)?

- ☐ Suspected COVID-19 infection
- ☐ Asthma
- ☐ Chronic obstructive pulmonary disease
- ☐ Pneumonia
- ☐ Common flu
- ☐ Heart attack
- ☐ Arrhythmias
- ☐ Other, please specify: ____

19. **Have you been to the emergency room or Urgent Care (when you were NOT admitted to the hospital overnight) since March 1st?**

- ☐ Yes, a UPMC hospital.
- ☐ Yes, NOT a UPMC hospital.
- ☐ No

19b. When did you most recently visit the emergency room or Urgent Care?

________ / ________ / ________
MM DD YYYY

19c. What was the main reason for your most recent emergency room or Urgent Care (you can look at the papers you received at discharge from the facility) ?

- ☐ Suspected COVID-19 infection
- ☐ Asthma
- ☐ Chronic obstructive pulmonary disease
- ☐ Pneumonia
- ☐ Common flu
- ☐ Heart attack
- ☐ Arrhythmias
- ☐ Other, please specify: ____

GAD7 Anxiety

Over the last month, how often have you been bothered by any of the following problems?

	Not at all ▼	Several days ▼	Over half the days ▼	Nearly every day ▼
**20a. Feeling nervous, anxious, or on edge**	0	1	2	3
**20b. Not being able to stop or control worrying**	0	1	2	3
**20c. Worrying too much about different things**	0	1	2	3
**20d. Trouble relaxing**	0	1	2	3
**20e. Being so restless that it's hard to sit still**	0	1	2	3
**20f. Becoming easily annoyed or irritable**	0	1	2	3
**20g. Feeling afraid as if something awful might happen**	0	1	2	3

PHQ-9 Depression

Over the last month, how often have you been bothered by any of the following problems?

	Not at all ▼	Several days ▼	Over half the days ▼	Nearly every day ▼
**21a. Little interest or pleasure in doing things**	0	1	2	3
**21b. Feeling down, depressed, or hopeless**	0	1	2	3
**21c. Trouble falling or staying asleep, or sleeping too much**	0	1	2	3
**21d. Feeling tired or having little energy**	0	1	2	3
**21e. Poor appetite or overeating**	0	1	2	3
**21f. Feeling bad about yourself — or that you are a failure or have let yourself or your family down**	0	1	2	3
**21g. Trouble concentrating on things, such as reading the newspaper or watching television**	0	1	2	3
**21h. Moving or speaking so slowly that other people could have noticed? Or the opposite — being so fidgety or restless that you have been moving around a lot more than usual**	0	1	2	3

ISI Sleep Survey

Please rate the severity of your sleep problems based on your life over the past month.

22a. Difficulty falling asleep:

A. None B. Mild C. Moderate D. Severe E. Very severe

22b. Difficulty staying asleep:

A. None B. Mild C. Moderate D. Severe E. Very severe

22c. Problem waking up too early:

A. None B. Mild C. Moderate D. Severe E. Very severe

22d. How satisfied/dissatisfied are you with your current sleep pattern?

A. Very Satisfied

B. Satisfied

C. Moderately satisfied

D. Dissatisfied

E. Very dissatisfied

22e. To what extent do you believe your sleep problem to interfere with your daily functioning (e.g. daytime fatigue, ability to function at work/daily chores, concentration, memory, mood, etc.) ?

A. Not at all interfering

B. A little

C. Somewhat

D. Much

E. Very much interfering

22f. How noticeable to others do you think your sleep problem is in terms of impairing the quality of your life?

A. Not at all noticeable

B. A little

C. Somewhat

D. Much

E. Very much noticeable

22g. How worried/distressed are you about your current sleep problem?

A. Not at all

B. A little

C. Somewhat

D. Much

E. Very much

22h. Over the past month (or since your last survey), on average, how many hours did you sleep per night? __________ hours per night

Recent Behavior

23. Over the <u>past month</u>, how many times have you visited a gym, restaurant, bar, or movie theater (not for takeout)? __________ times

24. Over the <u>past month</u>, how many times have you visited an event or gathering with more than 10 people? __________ times

25. Over the <u>past month</u>, approximately how many people outside of your household did you interact with or come within 6 feet of? __________ times

26. Over the <u>past month</u>, how often have you exercised more than 20 minutes (enough to breathe heavily and/or sweat)?

- ☐ Less than once per week
- ☐ About once per week
- ☐ More than once a week but less than 4 times a week
- ☐ More than 4 times a week
- ☐ Other

---

27. Since March 1st, have you experienced an interruption in follow-up with your provider who manages your Health care?

- ☐ Yes
- ☐ No

28. Since March 1st, have any ordered studies been delayed or canceled due to covid-19 restrictions?

- ☐ No
- ☐ Yes, chest imaging
- ☐ Yes, pulmonary function testing
- ☐ Yes, home oxygen evaluation
- ☐ Yes, sleep study
- ☐ Yes, other

29. Since March 1st, have you had any difficulty obtaining medications. If so, why?

- ☐ No
- ☐ Yes, medication not available at pharmacy
- ☐ Yes, difficulty getting prescription from physician
- ☐ Yes, difficulty picking up medication at pharmacy
- ☐ Yes, I was worried about going to the pharmacy to pick up my medications

30. Since March 1st, have you delayed seeking care for symptoms due to concerns related to covid-19 exposure?

☐ Yes
☐ No

31. Have you used any of the following virtual or telemedicine methods to receive care for a new health condition or for your ongoing medical care (not related to coronavirus)? Please select all that apply.

☐ Live, two-way video chat
☐ Phone call
☐ Text or other secure messaging
☐ Email
☐ I have been unable to use any virtual or telemedicine methods
☐ I have not tried to use any virtual or telemedicine methods

---

32. In response to the coronavirus outbreak, how worried do you feel about going to the doctor's office or hospital (including the emergency room) for necessary care not related to coronavirus symptoms?

☐ So worried I would avoid seeking the care I need
☐ Very worried
☐ Worried
☐ Moderately worried
☐ Somewhat worried
☐ Not worried

33. Have you traveled on an airplane since March 1st?

☐ Yes, within the month
☐ Yes, but only over a month
☐ No

34. Are you or one of your household members a healthcare worker (i.e., doctor, dentist, nurse, nurse's aide, paramedic, physician's assistant, home healthcare aid, hospital worker, pharmacist, or other type of healthcare worker)? Please select all that apply.

☐ I am, and I am currently working
☐ I am, but I am NOT currently working
☐ One of my household members is, and they are currently working
☐ One of my household members is, but they are NOT currently working
☐ No one in my household is a healthcare worker

35. Have you been in close contact with anyone (e.g., family members, friends, coworkers, acquaintances) who has experienced flu-like symptoms in the past month?

Close contact can include direct physical contact, face-to-face contact for longer than 15 minutes, exchange of bodily fluids, or being within 6 feet of the person for more than 15 minutes.

☐ Yes, within the last 10 days
☐ Yes, only over 10 days ago
☐ No
☐ I don't know/I'm not sure

36. Have you recently been in close contact with someone who was diagnosed with coronavirus?

Close contact can include direct physical contact, face-to-face contact for longer than 15 minutes, exchange of bodily fluids, or being within 6 feet of the person for more than 15 minutes.

- [ ] Yes, within the last 10 days
- [ ] Yes, only over 10 days ago
- [ ] No
- [ ] I don't know/I'm not sure

37. If social distancing guidelines and stay-at-home orders were lifted today, what steps would you feel comfortable taking?

- [ ] I would continue to stay-at-home or maintain social distancing behaviors
- [ ] I would continue or start to wear protective equipment (for example, a face mask) in public settings
- [ ] I would start to attend or host small social events (for example, going to a restaurant or having friends over for dinner), but would avoid large gatherings
- [ ] I would return to life as normal
- [ ] I am not currently following any social distancing guidelines or stay-at-home orders

38. How worried are you about getting coronavirus?

- [ ] Very Worried
- [ ] Worried
- [ ] Neutral
- [ ] Not very worried
- [ ] Not at all worried

39. How worried are you about someone in your household, friends or family getting coronavirus?

- [ ] Very Worried
- [ ] Worried
- [ ] Neutral
- [ ] Not very worried
- [ ] Not at all worried
- [ ] I live alone

40. Which of the following statements accurately reflects your feelings on the potential impact of the coronavirus on your own personal health?

- [ ] Coronavirus represents a **catastrophic** threat to my personal health
- [ ] Coronavirus represents a **major** threat to my personal health
- [ ] Coronavirus represents a **moderate** threat to my personal health
- [ ] Coronavirus represents a **minor** threat to personal my health
- [ ] Coronavirus represents an **insignificant** threat to my personal health

41. Which of the following statements accurately reflects your feelings on the potential impact of the coronavirus on you or your household's financial wellbeing?

- ☐ Coronavirus represents a **catastrophic** threat to my financial wellbeing
- ☐ Coronavirus represents a **major** threat to my financial wellbeing
- ☐ Coronavirus represents a **moderate** threat to my financial wellbeing
- ☐ Coronavirus represents a **minor** threat to my financial wellbeing
- ☐ Coronavirus represents an **insignificant** threat to my financial wellbeing

42. Have you practiced the following preventative behaviors in the past month in response to the coronavirus outbreak? Please select all that apply.

- ☐ Social-distanced or stayed-at-home (staying 6 feet away from individuals, limiting physical contact, and face-to-face interactions, leaving your home only for necessities)
- ☐ Worked from home
- ☐ Self-quarantined (staying in your home without leaving for any reason)
- ☐ Received a flu shot (flu vaccination)
- ☐ Focused on general health improvements (for example, exercise, sleep, eating healthy)
- ☐ None of the above

43. Which of the following disruptions to daily life caused by the coronavirus outbreak are you most worried about?

- ☐ Physical/mental health disruptions (for example, due to increased stress and lifestyle changes)
- ☐ Supply disruptions (for example, not having access to food or sanitation products)
- ☐ Medical disruptions (for example, not having access to healthcare)
- ☐ Social disruptions (for example, spending less time with friends/coworkers, people I care about getting sick)
- ☐ Financial disruptions (for example, increased costs, decreased income)
- ☐ Family disruptions (for example, homeschooling kids, family getting sick)
- ☐ Societal disruptions (for example, businesses closing, government powers, people suffering)

44. In the past month, have you worn any type of protective mask or face covering?

- ☐ Yes, every time I leave my home
- ☐ Yes, but only when I am in public settings where other social distancing measures are difficult to maintain (for example, at the grocery store)
- ☐ No

45. Over the past month, on average, how often have you washed or sanitized your hands?

- ☐ More than 10 times per day
- ☐ 5-10 times per day
- ☐ 2-4 times per day
- ☐ About once per day
- ☐ Less than once per day

46. Over the past month, on average has your diet changed?

- ☐ Yes, a lot
- ☐ Yes, somewhat
- ☐ Yes, but very little
- ☐ No, not at all

47. How many people live in your house with you

- ☐ one
- ☐ Two
- ☐ Three
- ☐ Four or more
- ☐ I live alone

48 How many pets do you have?

- ☐ One
- ☐ Two
- ☐ Three or more
- ☐ None

49. During the pandemic how much have you gone out of your home?

- ☐ Never
- ☐ Rarely
- ☐ For essentials only
- ☐ Often but less than normal
- ☐ Same as before
- ☐ I went out more than before the pandemic
